# Macrophage autophagy protects against hepatocellular carcinogenesis in mice

**DOI:** 10.1038/s41598-021-98203-5

**Published:** 2021-09-22

**Authors:** Anthony Deust, Marie-Noële Chobert, Vanessa Demontant, Guillaume Gricourt, Timothé Denaës, Allan Thiolat, Isaac Ruiz, Christophe Rodriguez, Jean-Michel Pawlotsky, Fatima Teixeira-Clerc

**Affiliations:** 1 INSERM U955, Institut Mondor de Recherche Biomédicale, Créteil, France; 2 Université Paris-Est, UMR-S955, Créteil, France; 3 Département de Virologie, Hôpital Henri Mondor, Créteil, France; 4 Plateforme de Génomique, Hôpital Henri Mondor, Créteil, France; 5 INSERM U955, Institut Mondor de Recherche Biomédicale, Hôpital Henri Mondor, 94000 Créteil, France

**Keywords:** Cancer microenvironment, Gastrointestinal cancer, Cancer, Cell biology, Immunology, Diseases

## Abstract

Autophagy is a lysosomal degradation pathway of cellular components that regulates macrophage properties. Macrophages are critically involved in tumor growth, metastasis, angiogenesis and immune suppression. Here, we investigated whether macrophage autophagy may protect against hepatocellular carcinoma (HCC). Experiments were performed in mice with deletion of the autophagy gene *Atg5* in the myeloid lineage (ATG5^Mye−/−^ mice) and their wild-type (WT) littermates. As compared to WT, ATG5^Mye−/−^ mice were more susceptible to diethylnitrosamine (DEN)-induced hepatocarcinogenesis, as shown by enhanced tumor number and volume. Moreover, DEN-treated ATG5^Mye−/−^ mice exhibited compromised immune cell recruitment and activation in the liver, suggesting that macrophage autophagy invalidation altered the antitumoral immune response. RNA sequencing showed that autophagy-deficient macrophages sorted from DEN mice are characterized by an enhanced expression of immunosuppressive markers. In vitro studies demonstrated that hepatoma cells impair the autophagy flux of macrophages and stimulate their expression of programmed cell death-ligand 1 (PD-L1), a major regulator of the immune checkpoint. Moreover, pharmacological activation of autophagy reduces hepatoma cell-induced PD-L1 expression in cultured macrophages while inhibition of autophagy further increases PD-L1 expression suggesting that autophagy invalidation in macrophages induces an immunosuppressive phenotype. These results uncover macrophage autophagy as a novel protective pathway regulating liver carcinogenesis.

## Introduction

Hepatocellular carcinoma (HCC), the most frequent primary malignant tumor of the liver, is among the most lethal and prevalent malignancies worldwide due to ineffective therapy and poor prognosis^1^. Both incidence and mortality of HCC are predicted to rise globally^2^. Since the introduction of the molecular targeted agent sorafenib in 2007, systemic therapy for HCC has changed drastically with the emergence of the combination therapy of immune checkpoint inhibitors and molecular targeted agents as the preferred option for first-line therapy (atezolizumab plus bevacizumab)^3^.

HCC commonly arises in patients with underlying chronic liver disease and is considered a typical inflammation-associated tumor. The molecular links between inflammation and hepatic carcinogenesis are not fully understood ; however, it is now well established that the inflammatory microenvironment plays a major role in tumor initiation and progression. Among the immune cells, macrophages are the most predominant leukocyte population in the tumor microenvironment and these cells have emerged as critical modulators of the tumor microenvironment, in opposition to their traditional function to eliminate cancer cells. Hepatic macrophages consisting of resident Kupffer cells and infiltrating monocytes are critically involved in the early stages of tumor initiation by releasing inflammatory mediators such as tumor necrosis factor-α (TNF-α), interleukin-6 (IL-6), and reactive oxygen species (ROS) that mediate DNA damage, oncogenic transformation and cancer-related inflammation. In established tumors, tumor-associated macrophages (TAMs) acquire a M2-like phenotype with poor antigen presentation capacity and increased macrophage density is strongly associated with a poor prognosis in patients with HCC^4^. These adverse effects may be partly explained by the ability of TAMs to promote tumor growth, to induce angiogenesis and metastasis and to dampen immune responses. In particular, TAMs produce various immunosuppressive mediators into tumor microenvironment that can suppress CD4 + and CD8 + T cell effector function. Additionally, TAMs also contribute to differentiation of regulatory T cells (Treg) with strong immune suppressive activities. Therefore, identification of the mechanisms by which macrophages are involved in the regulation of inflammation and the tumor microenvironment may pave the way for the identification of new potential therapeutic targets.

Macroautophagy (hereafter referred to as autophagy) is an evolutionarily conserved cellular degradation pathway of cytoplasmic components by lysosomes. During autophagy, cytoplasmic components are sequestered into a double-membrane vesicle, the autophagosome, which subsequently fuses with a lysosome for degradation. Autophagy occurs constitutively at basal levels to ensure clearance and recycling of long-lived or aggregated proteins and damaged organelles thus regulating cellular homeostasis. Autophagy is induced in response to various cellular stress, such as ROS, endoplasmic reticulum stress and nutrient deprivation. Defective autophagy has been implicated in a wide range of disorders including neurodegenerative diseases, infections, cardiovascular diseases and cancer^5^. In tumor cells, autophagy plays a complex role by acting both as a tumor suppressor by preventing oxidative stress and genomic instability and by allowing the survival of tumor cells exposed to environmental stresses (hypoxia, nutrient deprivation)^6^. The functions of tumor-associated stromal cells such as cancer-associated fibroblasts, endothelial cells and immune cells can also be modulated by autophagy. In macrophages, autophagy is a key regulator process that not only controls cell homeostasis but that is also implicated in the regulation of a wide range of specific immune functions that include monocyte to macrophage differentiation, clearance of intracellular pathogens, efferocytosis, antigen presentation, and reduction of the production of ROS and inflammatory cytokines. In this context, macrophage autophagy has recently emerged as an interesting therapeutic target regulating inflammation during chronic liver diseases, such as hepatitis, fibrosis and alcoholic and non-alcoholic liver diseases^7–11^.

In this study, we investigate the role of macrophage autophagy in hepatocarcinogenesis using a diethylnitrosamine (DEN)‐induced mouse HCC model. We found that mice with a specific knockdown of the autophagy-related gene 5 (Atg5) in the myeloid lineage (ATG5^Mye−/−^ mice) had an increased hepatocarcinogenesis and an altered antitumoral immune response compared to WT mice.

## Results

### Macrophage autophagy deficiency promotes DEN-induced HCC

**Figure 1:**
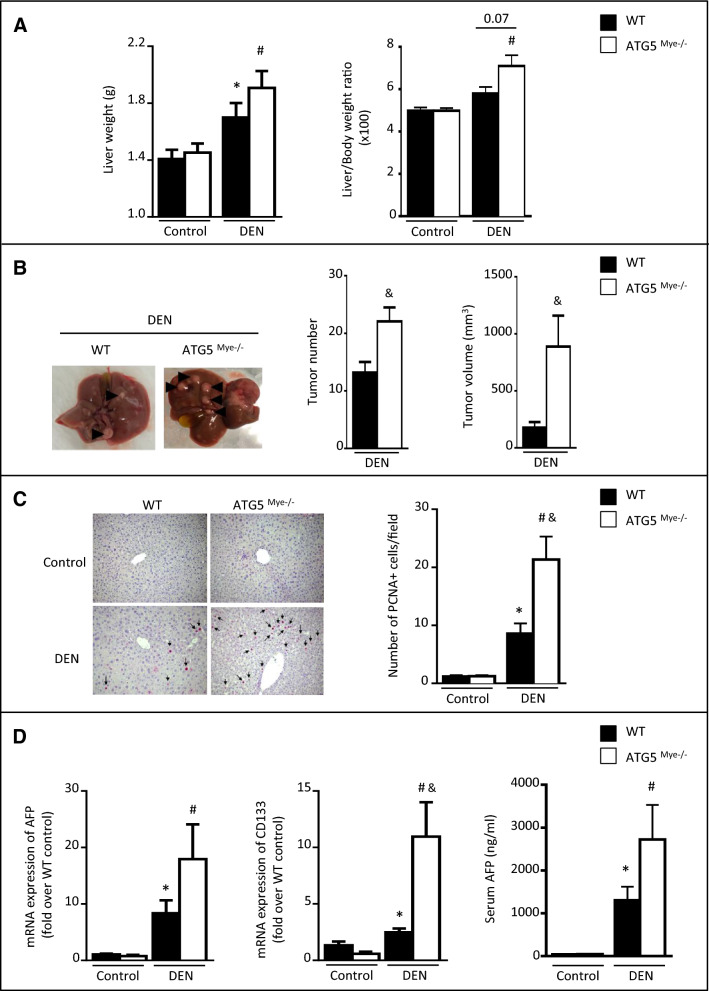
ATG5^Mye−/−^ mice show enhanced DEN-induced hepatocarcinogenesis. (**A**) Left, liver weight; Right, liver/body weight ratio of WT or ATG5^Mye−/−^ mice 10 months after DEN or saline solution administration. n = 28 for WT mice Control; n = 26 for ATG5^Mye−/−^ mice Control; n = 50 for WT mice DEN; n = 50 for ATG5^Mye−/−^ mice DEN. (**B**) Left, livers of WT or ATG5^Mye−/−^ mice 10 months after DEN treatment; Right, tumor number and tumor volume in WT or ATG5^Mye−/−^ mice 10 months after DEN treatment. n = 41 for WT mice DEN; n = 40 for ATG5^Mye−/−^ mice DEN. (**C**) Representative PCNA staining (magnification × 200) and quantification of PCNA-positive cell per field in WT or ATG5^Mye−/−^ mice 10 months after DEN or saline solution administration. n = 9 for WT mice Control; n = 6 for ATG5^Mye−/−^ mice Control; n = 10 for WT mice DEN; n = 10 for ATG5^Mye−/−^ mice DEN. (**D**) RT-PCR analysis of AFP and CD133 mRNA (left panel) and serum AFP levels (right panel) in WT or ATG5^Mye−/−^ mice 10 months after DEN or saline solution injection. n = 9 for WT mice Control; n = 6 for ATG5^Mye−/−^ mice Control; n = 21 for WT mice DEN; n = 26 for ATG5^Mye−/−^ mice DEN. Data are shown as mean ± SEM. *p < 0.05 for WT Control vs WT DEN, # for ATG5^Mye−/−^ Control vs ATG5^Mye−/−^ DEN and ^&^p < 0.05 for WT vs ATG5^Mye−/−^.

Most HCC develop in the context of severe liver fibrosis and cirrhosis caused by chronic liver inflammation and it is well established that cirrhosis is a major determinant of hepatocarcinogenesis. Because we have previously shown that liver fibrosis is exacerbated in mice invalidated for macrophage autophagy after chronic carbon tetrachloride treatment^7^, we sought to investigate the impact of macrophage autophagy invalidation on hepatocarcinogenesis independently of liver fibrosis in a model without fibrosis. Therefore, to address the role of macrophage autophagy in hepatocarcinogenesis, we exposed 15-day-old mice to a single injection of a low-dose of DEN (25 mg/kg). This model results in efficient HCC induction and bears genetic and histological similarities with human HCC that have a poor prognosis^12^. Tumor development was evaluated 10 months post DEN challenge. We observed enhanced liver weight and liver-to-body weight ratio (Fig. 1A) in ATG5^Mye−/−^ mice compared to WT mice. Moreover, we observed significantly higher tumor number and tumor volume (Fig. 1B) in ATG5^Mye−/−^ mouse livers compared to WT mice. Since excessive proliferation of hepatocytes is a hallmark of hepatocarcinogenesis^13^, we then analyzed the expression of proliferating cell nuclear antigen (PCNA) in the liver and found that this proliferation marker was significantly increased in ATG5^Mye−/−^ mice 10 months after DEN treatment compared to WT mice (Fig. 1C). Finally, the mRNA expression of the HCC markers, CD133 and alpha-fetoprotein (AFP) were enhanced in ATG5^Mye−/−^ mice compared to WT mice after DEN challenge suggesting that DEN-treated-ATG5^Mye−/−^ mice developed more aggressive HCC than their WT counterparts (Fig. 1D). The serum levels of AFP were also enhanced in ATG5^Mye−/−^ mice compared with WT mice after DEN treatment although differences are not statistically significant (Fig. 1D). These data demonstrate that macrophage autophagy deficiency increased DEN-induced hepatocarcinogenesis.

### Macrophage autophagy deficiency alters the antitumor immune response in the liver

**Figure 2:**
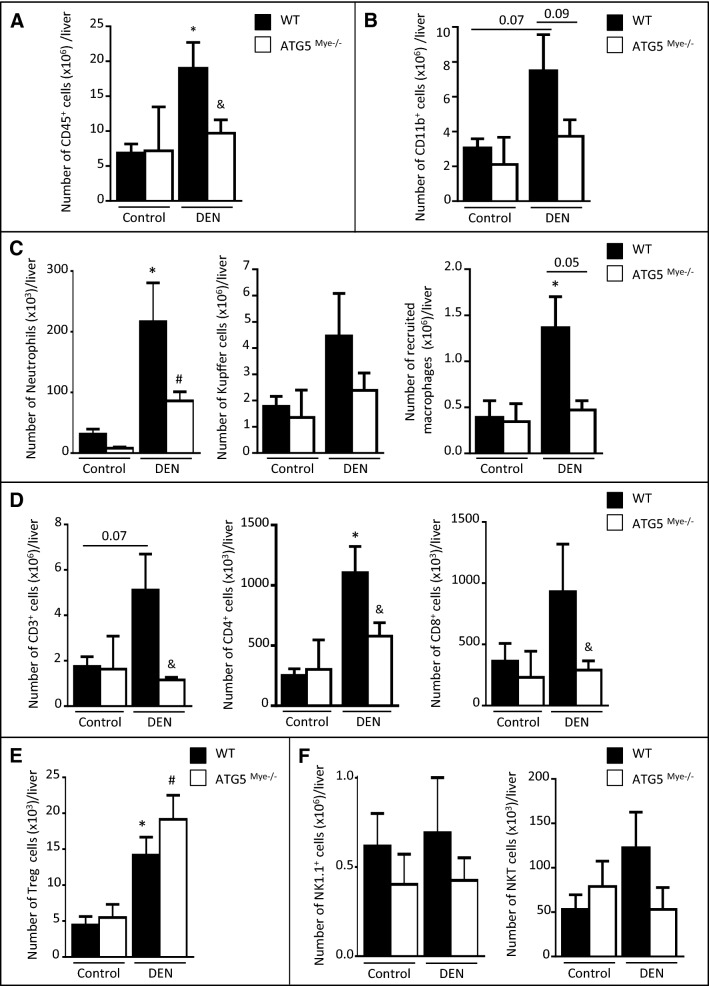
Macrophage autophagy deficiency alters the hepatic immune microenvironment after DEN challenge. Number of CD45^+^ cells (**A**), CD11b^+^ cells (**B**), neutrophils, Kupffer cells and recruited macrophages (**C**), CD3^+^, CD4^+^ and CD8^+^ T cells (**D**), Treg cells (**E**), NK cells and NKT cells (**F**) per liver in WT or ATG5^Mye−/−^ mice 10 months after DEN or saline solution administration. Data are shown as mean ± SEM. *p < 0.05 for WT Control vs WT DEN, # for ATG5^Mye−/−^ Control vs ATG5^Mye−/−^ DEN and ^&^p < 0.05 for WT vs ATG5^Mye−/−^. n = 6–14 for WT mice Control; n = 3–10 for ATG5^Mye−/−^ mice Control; n = 3–14 for WT mice DEN; n = 4–26 for ATG5^Mye−/−^ mice DEN.

It is now well established that autophagy plays a major role in the control of immune responses. To explore the immunological basis for enhanced hepatocarcinogenesis in the context of macrophage autophagy invalidation, we evaluated the impact of macrophage autophagy on the immune microenvironment during hepatocarcinogenesis. For this purpose, we characterized the different immune cell populations within the liver following DEN treatment by flow cytometry. Intrahepatic leukocytes (CD45^+^ cells) significantly accumulated in HCC livers compared to control livers in WT mice whereas no significant increase was observed in ATG5^Mye−/−^ mice (Fig. 2A). To identify the immune cell types decreased in the liver of ATG5^Mye−/−^ mice, we analyzed the distribution of innate and adaptive immune cells among hepatic immune cells. Total myeloid cell number tended to be increased in the liver of WT mice (p = 0.07) but not in ATG5^Mye−/−^ mice after DEN treatment (CD11b^+^ cells, Fig. 2B). Among myeloid cells, the number of neutrophils (Ly6G^+^ MHC II^-^ cells) was increased in both WT and ATG5^Mye−/−^ mice treated with DEN but was reduced in ATG5^Mye−/−^ mice as compared to their WT counterparts (Fig. 2C left). Finally, the number of monocyte‐derived macrophages (CD11b^hi^ F4/80^+^ cells) was increased in DEN-treated WT mice in contrast to DEN-treated ATG5^Mye−/−^ mice (Fig. 2C right) whereas the number of resident Kupffer cells (CD11b^+^ F4/80^hi^ cells) was not modified (Fig. 2C middle). ATG5^Mye−/−^ mice exposed to DEN also showed no increase in the number of lymphoid cells in response to DEN in contrast to their WT counterparts, particularly CD3^+^, CD4^+^ and CD8^+^ T lymphocytes (Fig. 2D). In contrast, the number of Treg cells (CD3^+^ CD4^+^ CD25^+^ FoxP3^+^ cells), a subset of immunosuppressive cells, tended to be higher in ATG5^Mye−/−^ mice as compared to WT mice after DEN treatment (Fig. 2E). Finally, the number of NK (CD19^-^ CD3^-^ NK1.1^+^ cells) and NKT (CD19^-^ CD3^+^ NK1.1^+^ cells) cells were not modified either by DEN treatment or macrophage autophagy invalidation (Fig. 2F). These results suggest that macrophage autophagy invalidation compromised the hepatocarcinogenesis-induced recruitment of immune cells into the liver.

**Figure 3:**
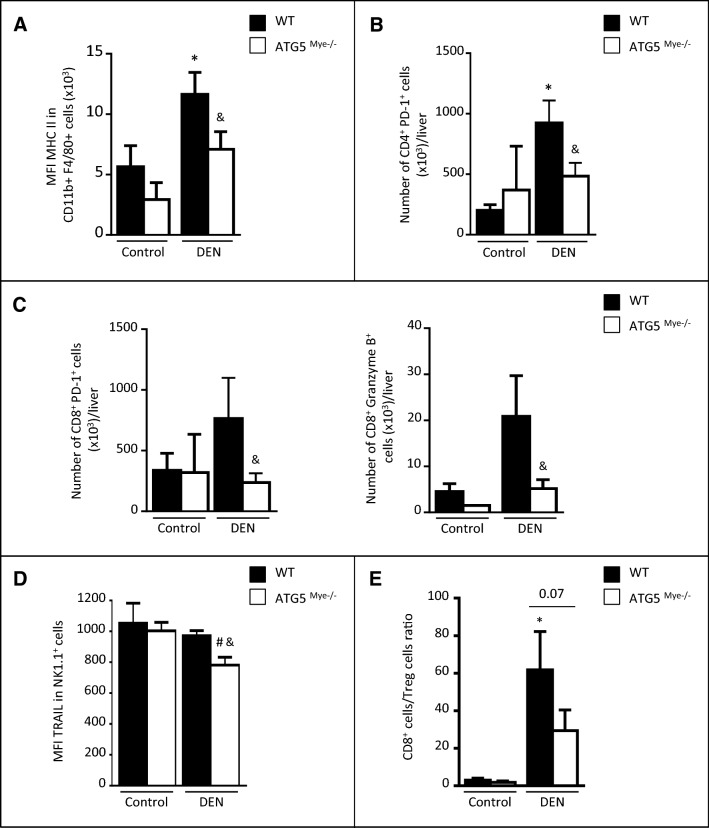
ATG5^Mye−/−^ mice display alteration in the hepatic immune cell activation. (**A**) MFI of MHC II in CD11b^+^ F4/80^+^ cells in WT or ATG5^Mye−/−^ mice 10 months after DEN or saline solution administration. (**B**) Number of CD4^+^ PD1^+^ T cells per liver in WT or ATG5^Mye−/−^ mice 10 months after DEN or saline solution administration. (**C**) Number of CD8^+^ PD1^+^ T cells and CD8^+^ Granzyme B^+^ cells per liver in WT or ATG5^Mye−/−^ mice 10 months after DEN or saline solution administration. (**D**) MFI of TRAIL in NK1.1^+^ cells in WT or ATG5^Mye−/−^ mice 10 months after DEN or saline solution administration. (**E**) CD8^+^ cells/Treg cells ratio in WT or ATG5^Mye−/−^ mice 10 months after DEN or saline solution administration. Data are shown as mean ± SEM. *, p < 0.05 for WT Control vs WT DEN, # for ATG5^Mye−/−^ Control vs ATG5^Mye−/−^ DEN and &, p < 0.05 for WT vs ATG5^Mye−/−^. n = 3–6 for WT mice Control; n = 4 for ATG5^Mye−/−^ mice Control; n = 6–10 for WT mice DEN; n = 6–13 for ATG5^Mye−/−^ mice DEN.

We next investigated the consequences of macrophage autophagy invalidation on immune cell activation. Hepatic macrophages from DEN-treated ATG5^Mye−/−^ mice showed reduced relative expression per cell of the maturation marker MCH class II as compared to their WT counterparts (Fig. 3A). In order to investigate whether this phenotype is associated with an impaired antitumor immune response, we analyzed the activation of lymphocytes and NK cells. The number of CD4^+^ PD-1^+^ T cells, CD8^+^ PD-1^+^ T cells and CD8^+^ Granzyme B + T cells were reduced in the liver of ATG5^Mye−/−^ mice after DEN treatment suggesting a reduced number of CD4^+^ and CD8^+^ effectors T cells in these mice (Fig. 3B,C). Moreover, NK cells from DEN-treated ATG5^Mye−/−^ mice showed reduced relative expression per cell of the cytotoxicity marker TRAIL (Fig. 3D). Finally, the ratio of CD8^+^ to Treg cells is reduced in ATG5^Mye−/−^ mice as compared to WT mice after DEN treatment suggesting that the tumoral microenvironment of DEN-treated ATG5^Mye−/−^ mice promoted Treg expansion over that of CD8^+^ T cells (Fig. 3E). Taken together, these data suggest that macrophage autophagy deficiency compromised the antitumor immune response.

### Transcriptome analysis of hepatic macrophages from HCC livers

Our data strongly suggested a reduced maturation of hepatic macrophages and an altered antitumor immune response in the liver of ATG5^Mye−/−^ mice after DEN treatment. We therefore analyzed the genetic program of hepatic macrophages by RNA sequencing to highlight mechanisms by which autophagy-deficient macrophages might mediate their deleterious impact. For this purpose, we sorted hepatic macrophages (CD45^+^ CD11b^+^ F4/80^+^ cells) from WT and ATG5^Mye−/−^ mice treated with DEN or saline solution and examined their transcriptome by RNA sequencing. Sorted cells expressed similar levels of macrophage-specific surface markers such as CD11b, F4/80 and CD68.

**Figure 4:**
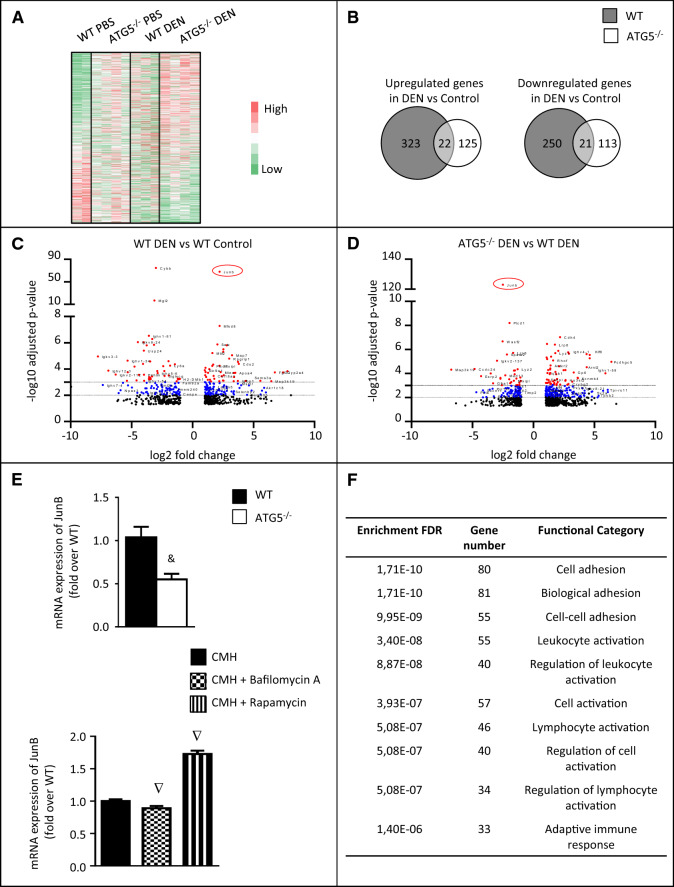
Sorted ATG5^−/−^ macrophages display a typical transcription program. (**A**) Heat map of the genes differentially expressed (using a threshold of twofold change and p value < 0.05) between WT and ATG5^−/−^ hepatic macrophages isolated from mice after control or DEN treatment. (**B**) Venn diagram for upregulated (left) and downregulated (right) genes in DEN vs Control WT or ATG5^−/−^ macrophages. (**C**) Volcano plot of the differentially expressed genes of WT macrophages sorted from DEN-treated mice as compared to WT macrophages sorted from control mice. (**D**) Volcano plot of the differentially expressed genes of ATG5^−/−^ macrophages sorted from DEN-treated mice as compared to WT macrophages sorted from DEN-treated mice. (**E**) RT-PCR analysis of JunB mRNA expression in peritoneal macrophages isolated from WT or ATG5^Mye−/−^ mice (upper panel) and in peritoneal macrophages exposed to the conditioned medium of Hepa1-6 cells (CMH) in the presence or absence of 100 nM bafilomycin A or 100 nM rapamycin. Data are shown as mean ± SEM of 6 samples per condition. ^&^p < 0.05 for WT vs ATG5^−/−^, ^∇^p < 0.05 for CMH vs CMH + bafilomycin A or CMH + rapamycin. (**F**) Gene ontology enrichment analysis show the top 10 modulated pathways by p-value enriched (false discovery rate, FDR) for genes significantly upregulated in ATG5^−/−^ macrophages isolated from DEN-treated mice. n = 3 for WT DEN, n = 4 for ATG5^−/−^ PBS, n = 3 for WT DEN; n = 4 for ATG5^−/−^ DEN.

RNA sequencing clearly indicated a typical transcriptional program in ATG5^−/−^ hepatic macrophages as shown by the heat map of the genes differentially expressed (using a threshold of twofold change and p value < 0.05) between WT and ATG5^−/−^ hepatic macrophages isolated from mice after control or DEN treatment (Fig. 4A). Moreover, differential gene expression analysis (using a threshold of twofold change and p value < 0.05) revealed that only 22 genes are simultaneously upregulated in both WT and ATG5^−/−^ macrophages while 323 genes and 125 genes were selectively induced in autophagy-sufficient or in autophagy-deficient macrophages, respectively, after DEN treatment (Fig. 4B, left panel). In addition, only 21 genes are simultaneously downregulated in both WT and ATG5^−/−^ macrophages while 250 genes and 113 genes were selectively downregulated in WT macrophages or in ATG5^−/−^ macrophages, respectively, after DEN treatment (Fig. 4B, right panel).

Volcano plots identified a number of differentially regulated genes in macrophages isolated from ATG5^Mye−/−^ mice treated with DEN compared to macrophages isolated from DEN-treated WT mice (Fig. 4C). Interestingly, one of these genes was JunB encoding a key transcription factor that controls both classical and alternative macrophage activation^14^ Indeed, macrophages sorted from DEN treated-WT mice were characterized by up-regulated JunB gene expression (log2 fold change 2.22, p-value 7.24 E−69) while the expression of JunB was significantly reduced in ATG5-deficient macrophages relative to its expression in ATG5-sufficient macrophages after DEN treatment (log2 fold change − 2.51, p-value 1.99 E−123) (Fig. 4C,D). These data prompt us to evaluate the impact of macrophage autophagy on Jun B mRNA expression. Compared to WT counterparts, ATG5^−/−^ mouse primary macrophages showed decreased expression of JunB (Fig. 4E upper panel). In keeping with these data, treatment of macrophages cultured in the presence of conditioned medium collected from the hepatoma cells, Hepa1-6, with the autophagy inhibitor bafilomycin A decreased the expression of JunB mRNA whereas, exposure of macrophages to the autophagy inducer rapamycin increased JunB mRNA expression (Fig. 4E bottom panel).

Gene ontology enrichment analysis indicated that genes significantly upregulated in ATG5^−/−^ macrophages isolated from DEN-treated mice displayed enrichment for pathways involved in cell adhesion, cell activation and adaptative immune response (Fig. 4F). The transcriptomic pattern suggested that ATG5^−/−^ macrophages isolated from DEN-treated mice exhibit an immunosuppressive phenotype with upregulation of M2 markers such as chitinase-like 1 (Chil1, log2 fold change 3.18, p-value 4.84 E−04) and Chil3 (log2 fold change 3.00, p-value 1.36 E−02). Furthermore, ATG5^−/−^ macrophages were enriched in the expression of a number of cytokines and cytokine receptors associated with immunoregulatory function of macrophages such as IL-13 (log2 fold change 2.89, p-value 8.52 E−03) and IL-9 receptor (IL-9R, log2 fold change 1.99, p-value 2.31 E−02). The expression of S100 calcium binding protein A8 (S100A8, log2 fold change 2.75, p-value 1.78 E−02) and S100A9 (log2 fold change 2.55, p-value 3.00 E−02), two damage-associated molecular pattern with pro-tumoral function was significantly induced in ATG5^−/−^ macrophages. We also identified additional pro-tumoral mechanisms by which ATG5^−/−^ macrophages may favor hepatocarcinogenesis, such as an increase in the expression of matrix metallopeptidase 8 (MMP8, log2 fold change 2.54, p-value 5.34 E−03)^14^, vascular endothelial growth factor A (VEGF-A, (log2 fold change − 1.91, p-value 1.67 E−02). Finally, ATG5^−/−^ macrophages show enhanced expression of the adenosine A2a receptor (ADORA2A, log2 fold change 1.03, p-value 2.07 E−02) which has been shown to suppress T and NK cell responses in the tumor microenvironment^14^.

All together, these data suggest that autophagy deficiency favors a typical transcriptional program in macrophages that may explain the compromised antitumor immune response and enhanced hepatocarcinogenesis in DEN-treated ATG5^Mye−/−^ mice.

### Macrophage autophagy deficiency alters tumor-induced splenic immune response

**Figure 5:**
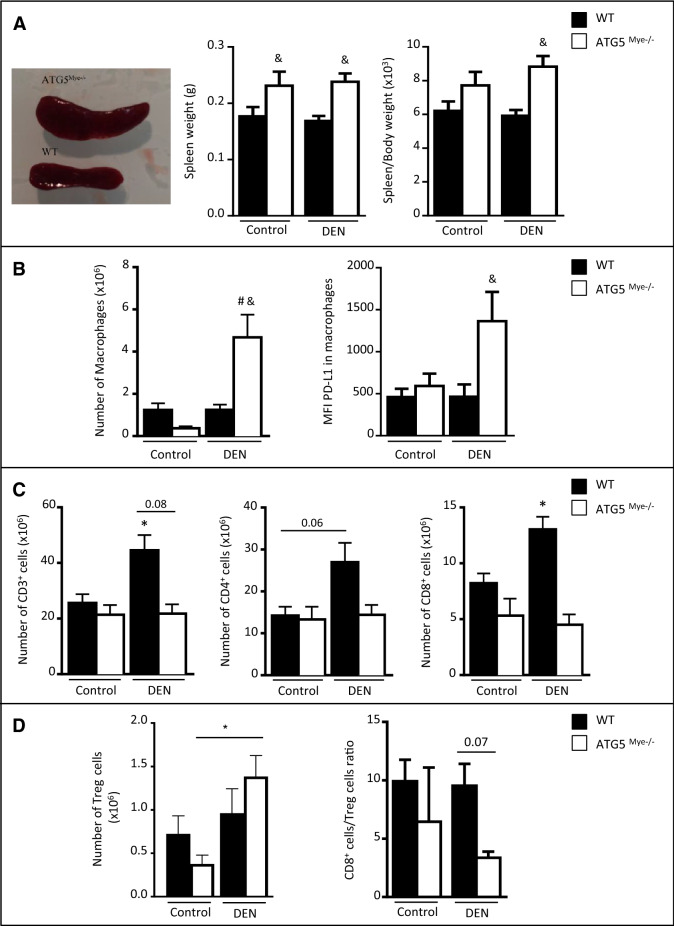
Macrophage autophagy invalidation affects the splenic immune response. (**A**) Left, spleens of WT or ATG5^Mye−/−^ mice 10 months after DEN treatment; Right, spleen weight and spleen/body weight ratio of WT or ATG5^Mye−/−^ mice 10 months after DEN or saline solution administration. (**B**) Number of splenic macrophages and MFI of PD-L1 in splenic macrophages in WT or ATG5^Mye−/−^ mice 10 months after DEN or saline solution administration. (**C**) Number of splenic CD3^+^, CD4^+^ and CD8^+^ T cells in WT or ATG5^Mye−/−^ mice 10 months after DEN or saline solution administration. (**D**) Number of splenic Treg cells and CD8 + cells/Treg cells ratio in WT or ATG5^Mye−/−^ mice 10 months after DEN or saline solution administration. Data are shown as mean ± SEM. *p < 0.05 for WT Control vs WT DEN, # for ATG5^Mye−/−^ Control vs ATG5^Mye−/−^ DEN and ^&^p < 0.05 for WT vs ATG5^Mye−/−^. n = 5–8 for WT mice Control; n = 4–5 for ATG5^Mye−/−^ mice Control; n = 8–12 for WT mice DEN; n = 4–7 for ATG5^Mye−/−^ mice DEN.

Our data demonstrating alterations of the intrahepatic immune profile in HCC livers of ATG5^Mye−/−^ mice prompted us to evaluate whether macrophage autophagy deficiency also affects immune cells in the spleen, an important lymphoid organ for priming immune effectors. Compared to WT mice, ATG5^Mye−/−^ mice showed enlarged spleens and an increase in spleen weight as well as in spleen-to-body weight ratio (Fig. 5A). In contrast to the liver, the number of macrophages (CD11b^+^ F4/80^+^ cells) is increased in the spleens of ATG5^Mye−/−^ mice in response to DEN challenge whereas no significant increase was observed in WT mice (Fig. 5B). However, these macrophages highly expressed the marker programmed cell death-ligand 1 (PD-L1) suggesting that they display an immunosuppressive phenotype (Fig. 5B). Finally, in agreement with the results obtained in the liver, CD3^+^, CD4^+^ and CD8^+^ T lymphocytes significantly accumulated in the spleens of DEN-treated WT mice compared to control mice whereas no significant increase was observed in ATG5^Mye−/−^ mice after DEN treatment (Fig. 5C). In contrast, the number of Treg cells is significantly enhanced in DEN-treated ATG5^Mye−/−^ mice whereas no significant increase was observed in WT mice after DEN treatment (Fig. 5D). Finally, the ratio of CD8^+^ to Treg cells is reduced in ATG5^Mye−/−^ mice as compared to WT mice after DEN treatment suggesting, as in the liver, that the tumoral microenvironment of DEN-treated ATG5^Mye−/−^ mice promoted Treg expansion over that of CD8^+^ T cells (Fig. 5D). These results demonstrate that macrophage autophagy deficiency altered the tumor-induced splenic immune response.

### Autophagy regulated PD-L1 expression by macrophages

**Figure 6:**
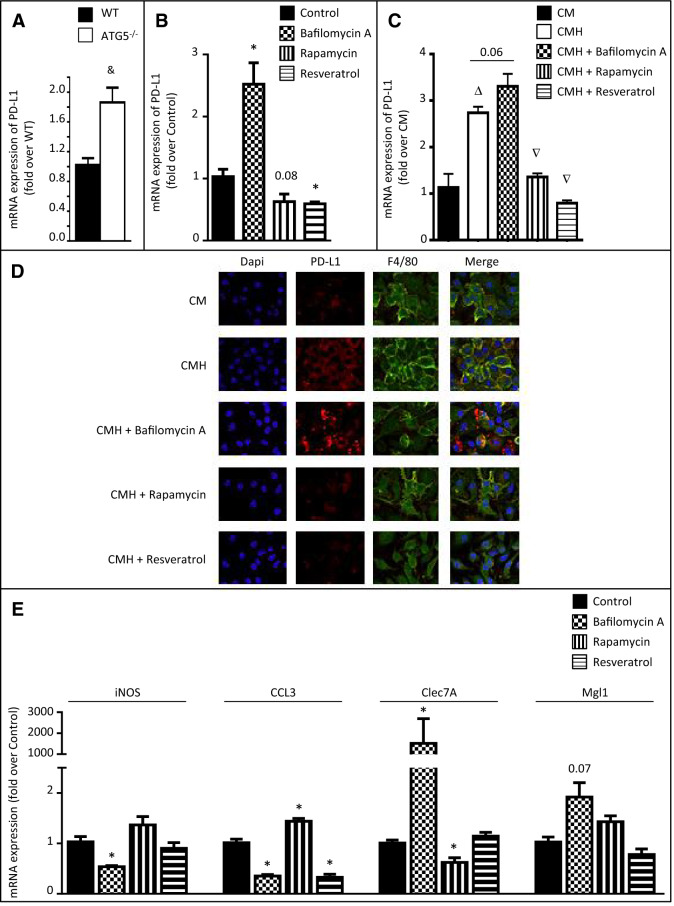
Autophagy regulates PD-L1 expression in macrophages and in TAMs. (**A**) RT-PCR analysis of PD-L1 mRNA in peritoneal macrophages isolated from WT or ATG5^Mye−/−^ mice. (**B**) RT-PCR analysis of PD-L1 mRNA in RAW264.7 cells in the presence or absence of 100 nM bafilomycin A, 100 nM rapamycin or 30 µM resveratrol. (**C**) RT-PCR analysis of PD-L1 mRNA in peritoneal macrophages exposed to the conditioned medium of Hepa1-6 cells (CMH) or to control medium (CM) in the presence or absence of 100 nM bafilomycin A, 100 nM rapamycin or 30 µM resveratrol. (**D**) Representative images of PD-L1 (red), F4/80 (green) and Dapi (blue) labeling in peritoneal macrophages exposed to control medium (CM) or to the conditioned medium of Hepa1-6 cells (CMH) in the presence or absence of 100 nM bafilomycin A, 100 nM rapamycin or 30 µM resveratrol (original magnification × 400). (**E**) RT-PCR analysis of iNOS, CCL3, Clec7A and Mgl1 mRNA in peritoneal macrophages in the presence or absence of 100 nM bafilomycin A, 100 nM rapamycin or 30 µM resveratrol. Data are shown as mean ± SEM of 6 samples per condition. ^&^p < 0.05 for WT vs ATG5^−/−^, *p < 0.05 for treatment vs control, ^Δ^p < 0.05 for CMH vs CM, ^∇^p < 0.05 for CMH vs CMH + bafilomycin A or CMH + rapamycin or CMH + resveratrol.

Among the mechanisms by which macrophages suppress tumor immunity are the expression of checkpoint molecules. One key molecule in this T cell suppressive pathway is PD-L1. Ligation of PD-L1 to its receptor PD-1 on T cells suppresses T cell activation and proliferation, and under normal conditions, functions to maintain peripheral tolerance. We have recently shown that PD-L1 expression by intratumoral inflammatory cells is related to tumor aggressiveness in HCC patients^14^. However, the mechanisms that regulate the expression of PD-L1 on TAMs are still poorly characterized. Our data showing that splenic macrophages from DEN-treated ATG5^Mye−/−^ mice highly express PD-L1 prompted us to evaluate the impact of autophagy on the expression of PD-L1 by mouse primary macrophages. Compared to WT counterparts, ATG5^−/−^ macrophages showed enhanced expression of PD-L1 (Fig. 6A). In keeping with these data, treatment of macrophages with the autophagy inhibitor bafilomycin A increased the expression of PD-L1 mRNA whereas, conversely, treatment of macrophages with autophagy inducers such as rapamycin or resveratrol reduced PD-L1 mRNA expression (Fig. 6B). We next investigated the expression of PD-L1 in the context of cancer by studying the impact of autophagy on the expression of PD-L1 by mouse primary macrophages cultured in the presence of conditioned medium collected from the hepatoma cells, Hepa1-6. As expected, the expression of PD-L1 was significantly enhanced in peritoneal macrophages exposed to conditioned medium collected from Hepa1-6 cells (CMH) as compared to control medium (CM) (Fig. 6C,D). The treatment of peritoneal macrophages exposed to Hepa1-6 conditioned medium with the autophagy inhibitor bafilomycin A further increased the expression of PD-L1 whereas, exposure of peritoneal macrophages to rapamycin or resveratrol prevented Hepa1-6 conditioned medium-induced increase in PD-L1 expression (Fig. 6C,D). These data demonstrate for the first time that autophagy regulates the expression of PD-L1 by macrophages.

Finally, we investigated whether the modulation of PD-L1 expression by autophagy is associated with a regulation of macrophage polarization. Treatment of macrophages with bafilomycin A decreased the expression of the M1 markers, iNOS and CCL3 while increasing that of the M2 markers Clec7A and Mgl1 (p = 0.07, Fig. 6E). Conversely, rapamycin increased the expression of the M1 marker CCL3 and decreased the expression of the M2 marker Clec7A (Fig. 6E). Finally, resveratrol only decreased the expression of the M1 marker CCL3 (Fig. 6E). Alltogether, these results suggest that macrophage autophagy favors the transition of macrophages towards an anti-tumor M1 phenotype.

### Tumor cells inhibited macrophage autophagy

**Figure 7:**
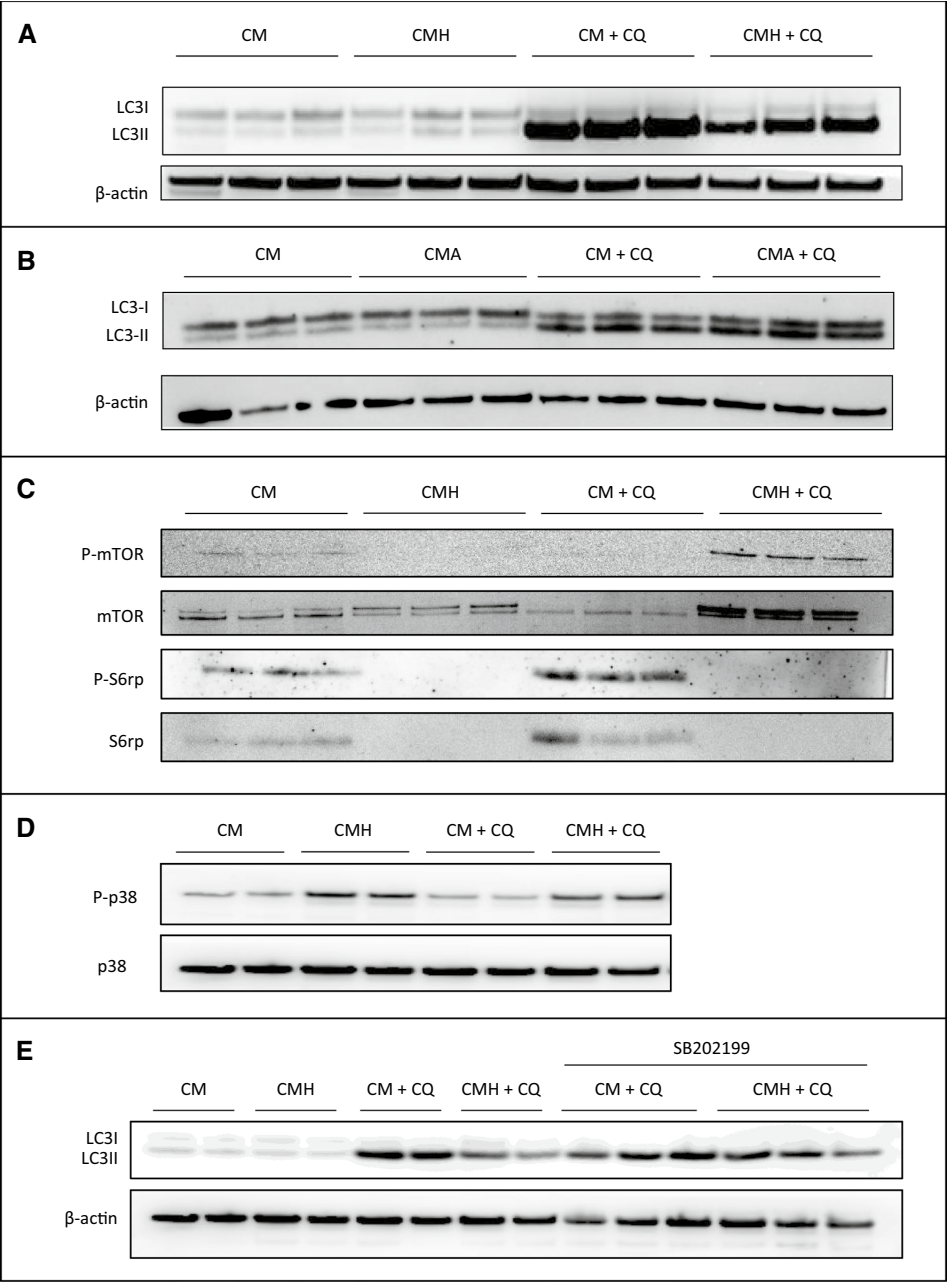
Tumor cells inhibit macrophage autophagy. (**A**) Representative western blot analysis of LC3 and β-actin in peritoneal macrophages exposed to the conditioned medium of Hepa1-6 cells (CMH) or to control medium (CM) in the presence or absence of 10 µM chloroquine (CQ). (**B**) Representative western blot analysis of LC3 and β-actin in peritoneal macrophages exposed to the conditioned medium of AML-12 cells (CMA) or to control medium (CM) in the presence or absence of 10 µM chloroquine (CQ). (**C**) Representative western blot analysis of P-mTOR, mTOR, P-S6rp and S6rp in peritoneal macrophages exposed to the conditioned medium of Hepa1-6 cells (CMH) or to control medium (CM) in the presence or absence of 10 µM chloroquine (CQ). (**D**) Representative western blot analysis of P-p38 and p38 in peritoneal macrophages exposed to the conditioned medium of Hepa1-6 cells (CMH) or to control medium (CM) in the presence or absence of 10 µM chloroquine (CQ). (**E**) Representative western blot analysis of LC3 and β-actin in peritoneal macrophages exposed to the conditioned medium of Hepa1-6 cells (CMH) or to control medium (CM) in the presence or absence of 10 µM chloroquine (CQ) and of 10 µM SB202199.

The ability of tumor cells to modulate the function of immune cells to promote cancer cell growth prompted us to evaluate whether tumor cells were able to inhibit autophagy in macrophages. Autophagy induction is associated with the conversion of the cytosolic form of microtubule-associated-protein light chain 3 (LC3-I) to the autophagosome-bound form of LC3 (LC3-II). Thus, the amount of LC3-II is directly correlated with the number of autophagosomes and is considered as a marker of the autophagic flux. We therefore first investigated whether tumor cells could regulate autophagy by studying LC3-ΙΙ expression by western blot in the presence of chloroquine (CQ), known to inhibit lysosomal degradation but not autophagosome formation. As shown in Fig. 7A, macrophages exposed to the conditioned medium of the hepatoma cells (CMH), Hepa1-6, showed a reduction in LC3-II accumulation in the presence of chloroquine as compared to macrophages cultured in control medium (CM). In contrast, autophagosome accumulation in the presence of chloroquine was similar between macrophages exposed to the conditioned medium of the hepatocyte cell line, AML-12 (CMA) and macrophages cultured in control medium (CM) (Fig. 7B). Autophagy flux was also monitored by quantification of the number of LC3-positive dots per cell, a marker of the number of autophagosomes. Culture of macrophages with the conditioned medium of the hepatoma cells reduced the number of LC3-positive dots both in basal condition and in the presence of chloroquine as compared to macrophages exposed to control medium (Fig. S2). These results demonstrated that macrophage autophagy inhibition is achieved by tumor cells but not by normal hepatocytes and suggest that macrophage autophagy inhibition could represent a mechanism by which tumor cells favor their own progression.

We have next investigated the signaling pathway involved in macrophage autophagy inhibition by hepatoma cells and first focused on mTOR which is a key factor that controls autophagy. We found that the phosphorylation of both mTOR and S6rp, an mTOR downstream effector, was decreased in peritoneal macrophages exposed to the conditioned medium of hepatoma cells (Fig. 7C). These data suggest that the mTOR pathway is not involved in macrophage autophagy inhibition by hepatoma cells. We next investigated the role of p38 MAPK which was shown to inhibit autophagy^15^. We showed that the conditioned medium of hepatoma cells induced the phosphorylation of p38 in basal condition and in the presence of chloroquine (Fig. 7D). Moreover, p38 inhibition by SB202199 restaured the conversion of LC3-I to LC3-II, indicating that enhanced activation of p38 leads to inhibition of macrophage autophagy by the conditioned medium of hepatoma cells (Fig. 7E).

## Discussion

Macrophages have emerged as critical players in HCC initiation and progression and the modulation of their functions has been proposed as an interesting therapeutic approach in cancer. In this context, elucidating the mechanisms underlying hepatocarcinogenesis and the contribution of macrophages to these processes is key to unravelling critical pathways in hepatocarcinogenesis and to rationally design therapeutic strategies targeting macrophages. In the present study, we provide the first demonstration that macrophage autophagy-controlled immune responses play a defense role against hepatocarcinogenesis and provide a rationale for modulating the activity of macrophage autophagy as part of HCC prevention and treatment.

A major finding of the present study is the identification of macrophage autophagy as a novel mechanism regulating the antitumor immune response. Indeed, our results demonstrate that during hepatocarcinogenesis, macrophage autophagy deficiency altered the intrahepatic immune responses as shown by the reduction in the number and the activation of hepatic macrophages, T lymphocytes and NK cells whereas the number of Treg tended to be higher in ATG5^Mye−/−^ mice as compared to WT mice. These data suggest that signals derived from autophagy-deficient macrophages affect the surrounding microenvironment. Here, by RNA sequencing of sorted hepatic macrophages from WT and ATG5^Mye−/−^ mice, we demonstrated that autophagy-deficient macrophages display a typical transcriptional program. In particular, ATG5^−/−^ macrophages isolated from DEN-treated mice show upregulation of genes associated with an immunosuppressive phenotype or which have been identified as major players in cancer development and progression such as Chil1, Chil3 and IL-13 as well as S100A8, S100A9, VEGF-A, MMP8 and Adenosine A2a receptor. Thus, it is well established that acquisition of an immunosuppressive phenotype by TAMs is associated with poor prognosis in HCC^16,17^, is capable of inhibiting the antitumor activity of effector T cells and NK cells^18^ and to promote the accumulation of Treg lymphocytes in the liver^19,20^. In agreement, our results show that hepatic macrophages from DEN-treated ATG5^Mye−/−^ mice showed reduced expression of the MCH class II as compared to their WT counterparts suggesting that these cells exhibit lower antigen-presenting capacity. In addition, analysis of PD1 and granzyme expression in CD8^+^ T cells suggests a decrease in their functionality. Finally, NK cells from DEN-exposed ATG5^Mye−/−^ mice are defective in their TRAIL-mediated killing pathway. Altogether, these data suggest that autophagy-deficient macrophages stimulate tumor growth by acting as immune suppressor cells of the innate and adaptive system and that modulating autophagy is an interesting strategy to enhance the antitumoral response of TAMs.

Another mechanism by which TAMs suppress antitumor immunity is the expression of immune checkpoint inhibitors that foster T cell exhaustion. In this regard, increased expression of PD-L1 by tumor-infiltrating macrophages is thought to inhibit effector T cells and correlates with increased tumor burden and reduced survival in HCC patients^21–24^. Moreover, we have recently shown that PD-L1 expression by intratumoral inflammatory cells is related to tumor aggressiveness in HCC patients^14^. However, the mechanisms that regulate the expression of PD-L1 on TAMs are still poorly characterized. Here, using in vitro studies, we clearly demonstrate for the first time that autophagy regulates PD-L1 expression in macrophages. Indeed, genetic invalidation of autophagy enhances the expression of PD-L1 mRNA. Moreover, pharmacologic inhibition of autophagy enhances the expression of PD-L1 on primary macrophages exposed to the conditioned medium of hepatoma cells whereas, conversely, activation of autophagy limits PD-L1 expression by macrophages. Moreover, pharmacologic inhibition of autophagy decreased the expression of M1 markers and increased that of M2 markers suggesting that macrophage autophagy inhibition favors the transition of macrophages towards a pro-tumor M2 phenotype. These data suggest that inhibition of macrophage autophagy could promote an immunosuppressive environment by inducing PD-L1 expression by these macrophages thereby altering the anti-tumor immune response. In order to translate these in vitro results into the in vivo context, we analyzed the expression of PD-L1 on hepatic and splenic macrophages because accumulation of cells with immunosuppressive activities is usually observed at both the tumor site and different primary and secondary lymphoid organs. Here, we show that splenic macrophages of DEN-exposed ATG5^Mye−/−^ mice strongly express PD-L1 and that hepatic macrophages show a non-significant increased expression of PD-L1 (data not shown). This can be explained by the fact that flow cytometry analysis have been performed on macrophages of the whole liver and not only on tumor-infiltrating macrophages. Whether the expression of PD-L1 is enhanced on tumor-infiltrating macrophages from ATG5^Mye−/−^ HCC livers is an issue that needs further investigation.

**Figure 8:**
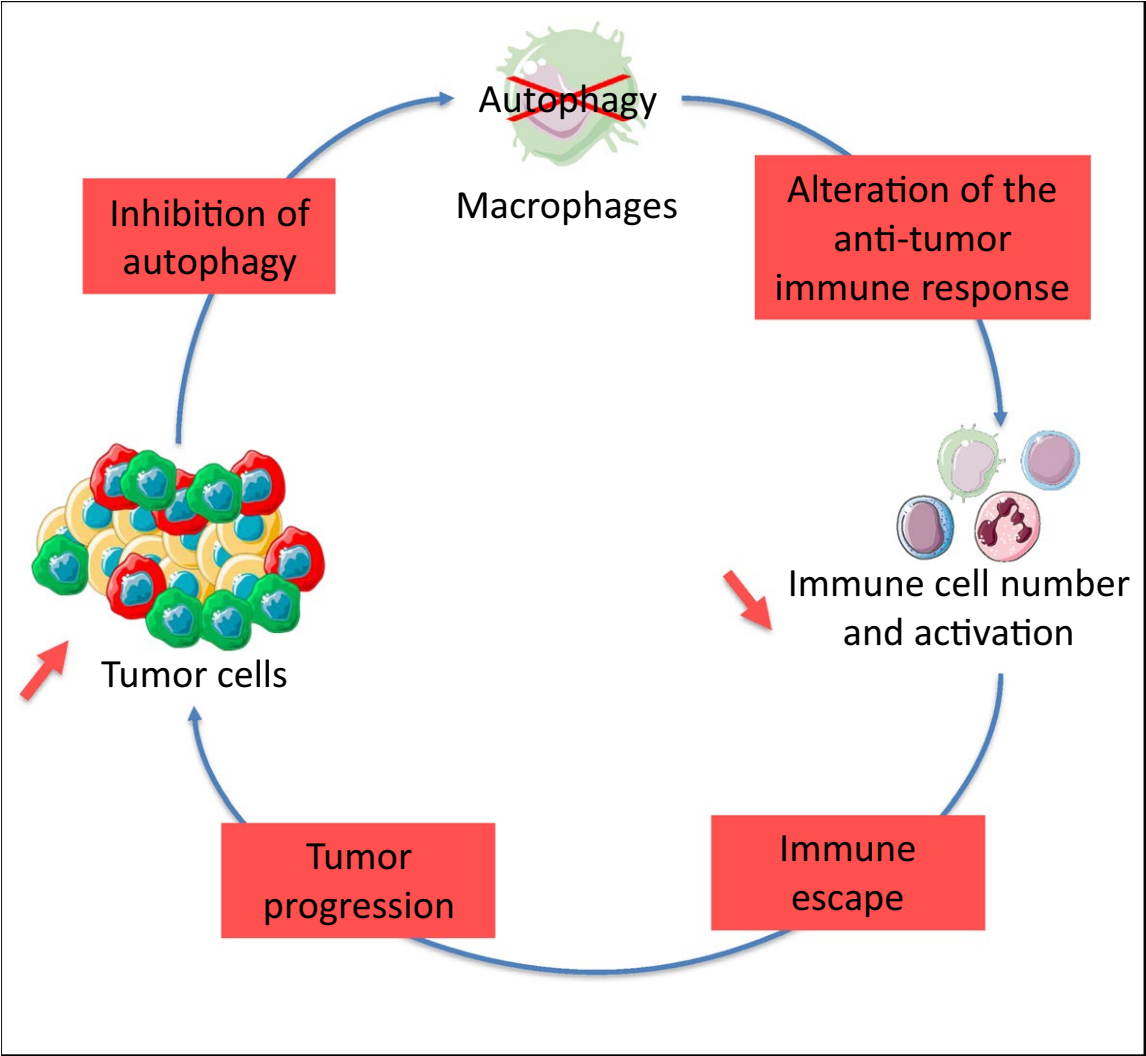
Schematic representation of the vicious circle in which inhibition of macrophage autophagy by tumoral cells could promote escape of HCC to antitumor immunity. Tumor cells inhibits macrophage autophagy, and macrophage autophagy inhibition alters the antitumor immune response, leading to HCC progression.

It is well established that the tumor microenvironment is essential for driving tumor progression by promoting cancer cell survival, migration, metastasis, and the ability to evade the immune system responses. In keeping with this concept, TAMs have been reported to not only play critical roles in cancer progression but also be educated or reprogrammed by the microenvironment itself^25^. Here, through in vitro studies, we report that tumor cells inhibit autophagy in macrophages. It is worthwhile to mention that this inhibition of macrophage autophagy is only achieved by hepatoma cells but not by normal hepatocytes. Moreover, our data show that tumor cells favor the hyporesponse of macrophages by inducing their expression of PD-L1, a major regulator of the immune checkpoint, thus altering the anti-tumor immune response. In addition, our data suggest that autophagy-deficient macrophages sorted from DEN-treated ATG5^Mye−/−^mice show upregulation of genes associated with an immunosuppressive phenotype. Altogether, these results suggest that macrophage autophagy inhibition could be a novel mechanism by which tumor cells favor their own progression by inducing an immunosuppressive environment. Moreover, these data suggest a vicious circle in which inhibition of macrophage autophagy by tumoral cells could promote escape of HCC to antitumor immunity and identify a crosstalk between tumor cells and macrophages (Fig. 8).

It is being recognized that macrophage autophagy displays a protective role in liver-related diseases namely acute liver injury, non-alcoholic and alcoholic liver disease and liver fibrosis^26,27^. Here, we demonstrate that macrophage autophagy protects against hepatocarcinogenesis. These results provide a rationale for inducing autophagy in macrophages as part of HCC prevention and treatment. However, in tumoral cells, autophagy has often been described as a “double-edged sword” since autophagy is a tumor-suppressive mechanism through tumor surveillance whereas it promotes cell survival under various stress conditions such as hypoxia, metabolic stress and cancer therapy. In this context, cell type-targeted strategies are a prerequisite to further consider autophagy as a potential target for cancer therapy.

In conclusion, our results suggest that macrophage autophagy favors hepatocarcinogenesis by inducing an immunosuppressive microenvironment. This data shed new light on the contribution of macrophages in liver cancer and on the crosstalk between tumor cells and macrophages. These data improve our understanding of the mechanisms underlying hepatocarcinogenesis and tumor escape and suggest that macrophage autophagy may represent a new therapeutic target for HCC.

## Methods

All methods were carried out in accordance with relevant guidelines and regulations.

### Animals and experimental design

#### Animals

Myeloid cell-specific Atg5 knockout (Atg5^fl/fl^ LysM-Cre^+/-^ or ATG5^Mye−/−^) mice were generated by crossing Atg5^flox/flox^ mice (kindly provided by Dr Noburo Mizushima, Japan) with transgenic mice expressing the Cre recombinase under the control of the lysozyme M promoter (LysMCre^+/+^ mice, Charles River). *Atg5*^flox/flox^ LysMCre^−/−^ mice were used as littermate WT controls. We have previously shown that Kupffer cells isolated from ATG5^Mye−/−^ mice exhibit autophagy dysfunction^7^. Animals were housed in pathogen-free animal facility and fed ad libitum*.* All the experiments were performed in accordance with relevant guidelines and regulations. All animal procedures were approved by the Committee for the Care and Use of Laboratory Animals of the Paris-Est Creteil University (ComEth) and The French Ministry of Higher Education, Research and Innovation (Authorization N°05,344.02). All animal work was performed in accordance with Animal Research: Reporting of In Vivo Experiments (ARRIVE) guidelines and regulations.

#### Hepatocarcinogenesis model

DEN is widely used to study inflammation-mediated hepatocellular carcinogenesis since DEN is a genotoxic agent that generates oxidative stress that damages DNA and induces hepatocyte death and cytokine-driven compensatory proliferation, acting as a tumor promoter like in patients^28^ 15-day-old mice received an intraperitoneal injection of DEN (25 mg/kg) in saline solution and were sacrificed after 10 months. Control animals received saline solution. The tumor volume was measured using the formula V = 4/3πr^3^ where r is the radius of the tumor.

### Cell isolation and culture

#### Hepatic non-parenchymal cell (NPC) isolation

Livers were perfused through the portal vein with a 30 μg/ml liberase solution (Roche), mechanically disrupted, and digested for 10 min at 37 °C in a 30 μg/ml liberase solution with 10 μg/ml of DNAse I (Roche) and 200 μg/ml pronase (Roche), then filtered through a 100-μm cell strainer. Parenchymal cells were separated from NPC by centrifugation for 5 min at 60×*g*. The supernatant was collected and centrifuged for 10 min at 420×*g*. The pellet containing the NPC was resuspended in a 40% percoll solution (GE Healthcare), laid onto an 80% percoll solution. After centrifugation for 20 min at 800×*g* at room temperature, the NPC fraction was collected at the interface and subjected to flow cytometric analysis or cell sorting.

#### Splenocyte isolation

Splenocytes were isolated from the spleen by mechanical disruption in phosphate-buffered saline (PBS).

#### Cell lines

RAW264.7 (TIB-71), AML-12 (CRL-2554) and Hepa1-6 (CRL-1830) were obtained from ATCC and were cultivated at 37 °C with 5% CO2.

#### Peritoneal macrophages

Peritoneal macrophages were harvested by lavage with PBS (Life Technologies), 3 days after injection of 1.5 ml of sterile 4% thioglycolate medium (BD Biosciences) into the peritoneal cavity of the mice. After red blood cells lysis with Red Blood Cell Lysis solution (Miltenyi Biotec), cells were seeded in RPMI supplemented with 10% fetal bovine serum (FBS) and washed after 3 h of adhesion. Conditioned medium experiments were performed by adding centrifuged conditioned medium obtained from AML-12 or Hepa1-6 cells for 6 h. When indicated, cells were treated with 10 μM chloroquine (Sigma), 100 nM bafilomycin A (Enzo Life Sciences), 100 nM rapamycin (Sigma) or 30 μM resveratrol (TCI). When indicated, cells were treated with 10 μM SB202199 in the presence of control medium or conditioned medium from Hepa-1.6 cells for 6 h.

### Flow cytometry analysis and cell sorting

After red blood cell lysis, stainings were performed in the presence of 10 μl/mL of FcR blocking reagent (Miltenyi) in PBS with 2% FBS. For flow cytometry analysis, acquisition and data analysis were conducted on a LSR Fortessa cytometer (BD Biosciences) and FlowJo v10.7 software. For Next Generation Sequencing, cells were sorted using Influx Cell Sorter (BD Biosciences) and BD FACSTM v1.2.0.142 software (BD Biosciences). We used the following antibodies from BD Biosciences or eBiosciences: CD11b-PE-Cy7, CD19-PE-Texas Red, CD25-APC, CD253-APC, CD274-PE, CD3e-PE-Cy7, CD4-efluor 450, CD45-BV711, CD8b-FITC, F4/80-Alexa-eFluor 647, FoxP3-PE, Granzyme B-PE, Ly6G-FITC, MHC Class II-APC efluor 780, NK1.1-FITC, PD1-APC. Live and dead cells were distinguished using the LIVE/DEAD Fixable Aqua Dead Cell Stain Kit (Life technologies). The gating strategy is shown in Fig. S1.

### Library preparation, sequencing and data analyses

RNA was assessed on a Tapestation (Agilent). DNA libraries were prepared according to the manufacturer’s instructions (Illumina). Each library was sequenced on the Illumina NextSeq 500 device and a High Output 75 cycles cartridge with a capacity of 400 million readings. Expression analysis was conducted following these guidelines (https://doi.org/10.1038/nprot.2016.095). Reads were first mapped to the mouse genome (GCF_000001635, v24) using Hisat2 (2.1.0) (https://doi.org/10.1038/nmeth.3317), counted as reads per gene with Stringtie (1.3.5) (https://doi.org/10.1038/nbt.3122), and then analyzed using the statistical algorithm DESeq2 (1.22.2) (https://doi.org/10.1186/s13059-014-0550-8). All samples used for host transcriptome analysis have at least 1,379,285 transcripts per sample with an average of 1,773,354 accross all samples. Wald test was used to test significance expression and Benjamini and Hochberg procedure let to adjust p-value. Gene Ontology Enrichment Analysis was performed with ShinyGO (v0.61).

### Immunohistochemistry

Immunostaining for PCNA antigen was performed on paraffin-embedded liver tissue sections (4 μm thick) with a mouse monoclonal anti-PCNA antibody (1:50; Santa Cruz Biotechnology) and the MOM immunodetection kit (Vector, PK2002) according to the manufacturer’s instructions. The number of PCNA-positive cells were quantified from 10 fields (× 200 magnification) from 6 to 10 mice/group. No staining was observed when omitting the primary antibody.

### Immunocytochemistry

Immunocytochemical detection was carried out as previously decsribed^7^ with an anti-LC3 antibody (1:200, clone 5F10; Nanotools, 0231-100), an anti-PD-L1 antibody (eBiosciences 14-5982-82), an anti-F4/80 antibody Alexa Fluor 647 (Invitrogen MF48021) and with the following secondary antibodies: goat anti-mouse Alexa 555 (Life Technologies, A21424 or goat anti-rabbit Alexa Fluor 555 (Life Technologies, A31629). No staining was observed when the primary antibody was omitted. The number of LC3-puncta per cell was quantified in a minimum of 200 cells/condition.

### Western blot analysis

Western blot analysis was carried out as previously decsribed^7^ with the antibodies as follow: anti-LC3b (1:500, Novus Biologicals), rabbit anti-mTOR (Cell signaling 2972), rabbit anti-P-mTOR (Cell signaling 5536), rabbit anti-rp-S6 (Cell signaling 2217), rabbit anti-P-S6rp (Cell signaling 5364), rabbit anti-P-p38 (Cell signaling 9211), rabbit anti p-38 (Cell signaling 9212) and mouse monoclonal anti-β actin (1:10,000; Sigma) and with appropriate conjugated secondary antibodies (Jackson ImmunoResearch).

### Immunoassay

AFP was quantified by Enzyme Linked Immunosorbant Assay (ELISA, R&D Systems, MAFP00) on serum according to manufacturer’s instructions. Cytokines were quantified by ELISA (Invitrogen ref 88-7314-22 for IFN-γ and Invitrogen 88-7064-22 for IL6) on culture supernatants from peritoneal macrophages according to manufacturer's instructions.

### RNA preparation and real time polymerase chain reaction

Total RNA was extracted using the RNeasy Mini kit (Qiagen, 7406). 2 μg of total RNA was reverse transcribed using the High Capacity cDNA Reverse Transcription Kit (Life Technologies, 4368813). The resulting cDNA was subjected to real time polymerase chain reaction (RT-PCR) on a LightCycler 480 system (Roche Diagnostics), using the QuantiTect SYBR Green PCR kit (Qiagen, 204,143). The primer sequences were from Eurofins Genomics and as follows: 18S, F: 5′-AACTTTCGATGGTAGTCGCCGT-3′, R: 5′-TCCTTGGATGTGGTAGCCGTTT -3′; AFP, F: 5′-TGACAACAAGGAGGAGTGCTTCCA-3′, R: 5′-AATGGTTGTTGCCTGG AGGTTTCG-3′; CCL3, F: 5’-TGAGAGTCTTGGAGGCAGCGA-3’, R: 5’-TGTGGCTACTTGGCAGCAAACA-3’ ; CD133, F: 5′-CCCTCCAGCAAACAAGCAAC-3′, R: 5′- ACAGCCGGAA GTAAGAGCAC-3′; Clec7A, F: 5’-AGTGAAGGGCCATGGTTCTG-3’, R: 5’-GTTCCTTCTCACAGATACTG-3’; iNOS, F: 5’-AATCTTGGAGCGAGTTGTGG-3’, R: 5’-CAGGAAGTAGGTGAGGGCTTG-3’; JunB, F: 5’-CCTTTCTATCACGACGACTC-3’, R: 5’-TAGTCGTGTAGAGACAGGCT-3’; Mgl1, F: 5’-TGGCCTGAAGCTGACAAGTA-3’, R: 5’-AGGCCGATCCAACTAACCACATT-3’. PD-L1, F: 5’-CAACACATCCTCCACAGAAC-3’, R: 5’-CGCCACA TTTCTCCACATCT-3.

### Statistical analysis

Data are expressed as means ± standard error of the mean (SEM). Statistical analysis was performed by Mann–Whitney test using Prism 5.0 software (GraphPad). *p* < 0.05 was considered statistically significant.

## Supplementary Information

Supplementary Information.

## References

[1] Kulik L, El-Serag HB (2019). Epidemiology and management of hepatocellular carcinoma. Gastroenterology.

[2] Valery PC (2018). Projections of primary liver cancer to 2030 in 30 countries worldwide. Hepatology.

[3] Kudo M (2020). Recent advances in systemic therapy for hepatocellular carcinoma in an aging society: 2020 Update. Liver Cancer.

[4] Ding T (2009). High tumor-infiltrating macrophage density predicts poor prognosis in patients with primary hepatocellular carcinoma after resection. Hum. Pathol..

[5] Jiang P, Mizushima N (2014). Autophagy and human diseases. Cell Res..

[6] White E (2015). The role for autophagy in cancer. J. Clin. Invest..

[7] Lodder J (2015). Macrophage autophagy protects against liver fibrosis in mice. Autophagy.

[8] Liu K (2015). Impaired macrophage autophagy increases the immune response in obese mice by promoting proinflammatory macrophage polarization. Autophagy.

[9] Denaes T (2016). The cannabinoid receptor 2 protects against alcoholic liver disease via a macrophage autophagy-dependent pathway. Sci. Rep..

[10] Ding WX, Jaeschke H (2016). Autophagy in macrophages regulates the inflammasome and protects against liver injury. J. Hepatol..

[11] Ilyas G (2016). Macrophage autophagy limits acute toxic liver injury in mice through down regulation of interleukin-1beta. J. Hepatol..

[12] Lee JS (2004). Application of comparative functional genomics to identify best-fit mouse models to study human cancer. Nat. Genet..

[13] Qin LX, Tang ZY (2002). The prognostic molecular markers in hepatocellular carcinoma. World J. Gastroenterol..

[14] Calderaro J (2016). Programmed death ligand 1 expression in hepatocellular carcinoma: Relationship With clinical and pathological features. Hepatology.

[15] He Y, She H, Zhang T, Xu H, Cheng L, Yepes M, Zhao Y, Mao Z (2018). p38 MAPK inhibits autophagy and promotes microglial inflammatory responses by phosphorylating ULK1. J. Cell Biol..

[16] Budhu A (2006). Prediction of venous metastases, recurrence, and prognosis in hepatocellular carcinoma based on a unique immune response signature of the liver microenvironment. Cancer Cell.

[17] Dong P (2016). CD86(+)/CD206(+), diametrically polarized tumor-associated macrophages, predict hepatocellular carcinoma patient prognosis. Int. J. Mol. Sci..

[18] Wu Y (2013). Monocyte/macrophage-elicited natural killer cell dysfunction in hepatocellular carcinoma is mediated by CD48/2B4 interactions. Hepatology.

[19] Hefetz-Sela S (2014). Acquisition of an immunosuppressive protumorigenic macrophage phenotype depending on c-Jun phosphorylation. Proc. Natl. Acad. Sci. U S A.

[20] Zhou J (2009). Increased intratumoral regulatory T cells are related to intratumoral macrophages and poor prognosis in hepatocellular carcinoma patients. Int. J. Cancer.

[21] Petty AJ, Yang Y (2017). Tumor-associated macrophages: Implications in cancer immunotherapy. Immunotherapy.

[22] Prieto J, Melero I, Sangro B (2015). Immunological landscape and immunotherapy of hepatocellular carcinoma. Nat. Rev. Gastroenterol. Hepatol..

[23] Kuang DM (2009). Activated monocytes in peritumoral stroma of hepatocellular carcinoma foster immune privilege and disease progression through PD-L1. J. Exp. Med..

[24] Wu K (2009). Kupffer cell suppression of CD8+ T cells in human hepatocellular carcinoma is mediated by B7–H1/programmed death-1 interactions. Cancer Res..

[25] Biswas SK, Allavena P, Mantovani A (2013). Tumor-associated macrophages: Functional diversity, clinical significance, and open questions. Semin. Immunopathol..

[26] Mallat A (2014). Autophagy: A multifaceted partner in liver fibrosis. Biomed. Res. Int..

[27] Allaire M (2019). Autophagy in liver diseases: Time for translation?. J Hepatol.

[28] Verna L, Whysner J, Williams GM (1996). N-nitrosodiethylamine mechanistic data and risk assessment: Bioactivation, DNA-adduct formation, mutagenicity, and tumor initiation. Pharmacol. Ther..

