# Supplementary material for: Macrophage autophagy protects against hepatocellular carcinogenesis in mice
Source: Sci Rep. 2021 Sep 22;11:18809. doi: 10.1038/s41598-021-98203-5 (PMC8458469; doi:10.1038/s41598-021-98203-5)
Supplement: Supplementary file 1 — Supplementary Information. [file 41598_2021_98203_MOESM1_ESM.pdf]

MACROPHAGE AUTOPHAGY PROTECTS AGAINST HEPATOCELLULAR CARCINOGENESIS IN MICE

Anthony Deust, Marie-Noële Chobert, Vanessa Demontant, Guillaume Gricourt, Timothé Denaës, Allan Thiolat, Isaac Ruiz, Christophe Rodriguez, Jean-Michel Pawlotsky, Fatima Teixeira-Clerc

Supplementary Figures

Figure S1: Gating strategies used in flow cytometry analysis. Gating strategy for (A) CD45+ cells, CD11b+ cells, Kupffer cells, recruited hepatic macrophages and neutrophiles, (B) CD3+, CD4+ and CD8+ T cells, (C) Treg cells, (D) NK and NKT cells.

Figure S2. Left, representative images of LC3 (red), F4/80 (green) and Dapi (blue) labeling in peritoneal macrophages exposed to the conditioned medium of Hepa1-6 cells (CMH) or to control medium (CM) in the presence or absence of 10 µM chloroquine (CQ) (original magnification x100). Right, quantification on the number of LC3 dots/cell in peritoneal macrophages exposed to the conditioned medium of Hepa1-6 cells (CMH) or to control medium (CM) in the presence or absence of 10 µM chloroquine (CQ). Data are shown as mean ± SEM. *, $p<0.05$ for CMH vs CM ; &, $p<0.05$ for CQ vs vehicle.

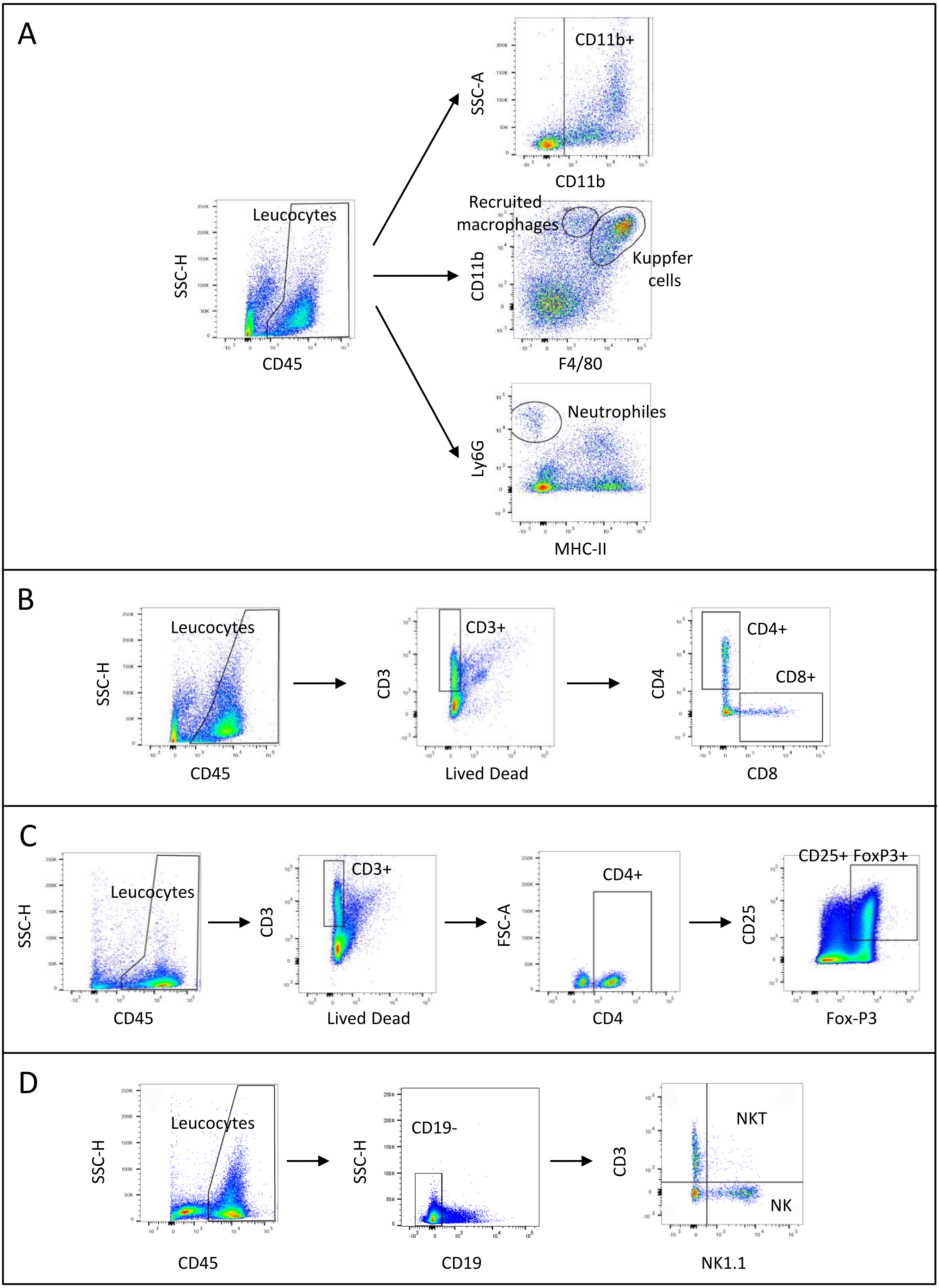
A
CD11b+
SSC-A
CD11b
Recruited
macrophages
Leucocytes
SSC-H
CD11b
Kuppfer
cells
CD45
F4/80
Neutrophiles
Ly6G
MHC-II
B
Leucocytes
SSC-H
CD45
CD3+
CD3
Lived Dead
CD4+
CD8+
CD4
CD8
C
Leucocytes
SSC-H
CD45
CD3+
CD3
Lived Dead
CD4+
FSC-A
CD4
CD25+ FoxP3+
CD25
Fox-P3
D
Leucocytes
SSC-H
CD45
CD19-
SSC-H
CD19
NKT
NK
CD3
NK1.1

Figure S1

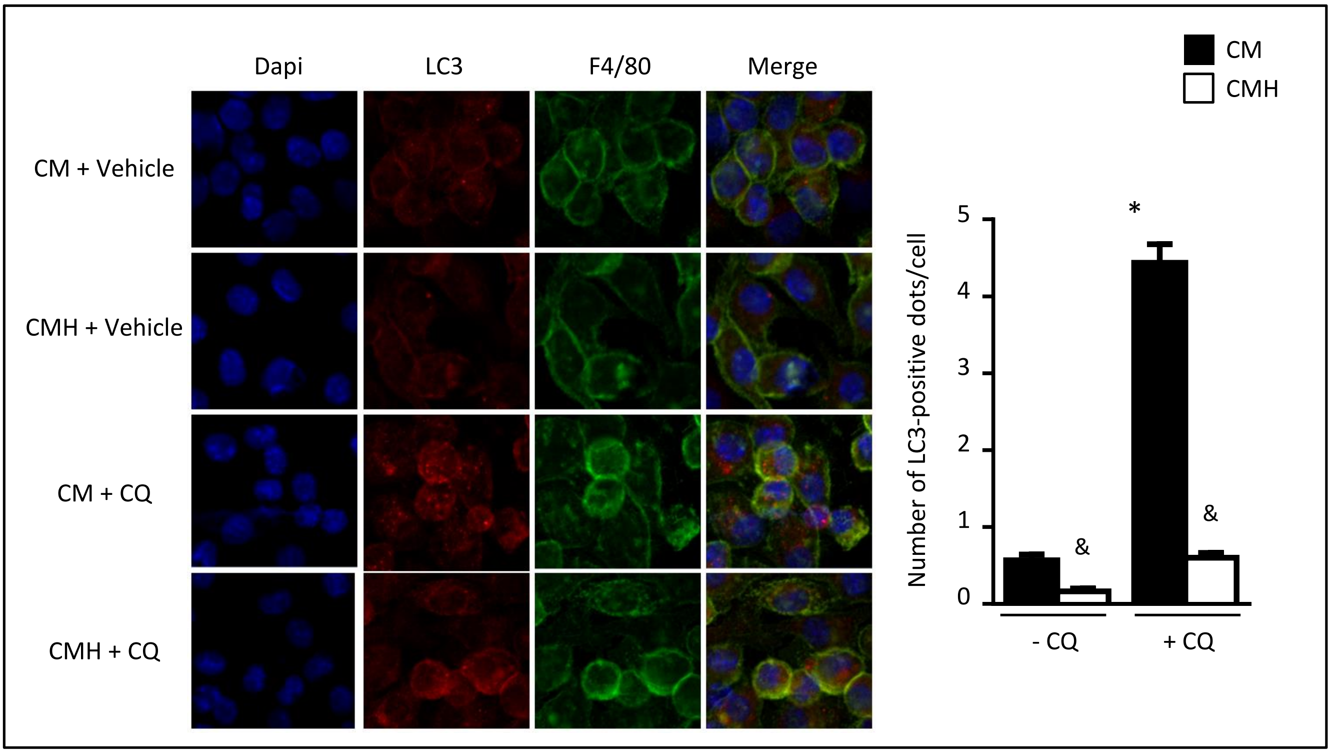
Dapi
LC3
F4/80
Merge
CM + Vehicle
CMH + Vehicle
CM + CQ
CMH + CQ
CM
CMH
Number of LC3-positive dots/cell
0
1
2
3
4
5
*
&
&
- CQ
+ CQ

Figure S2

Figure 7A

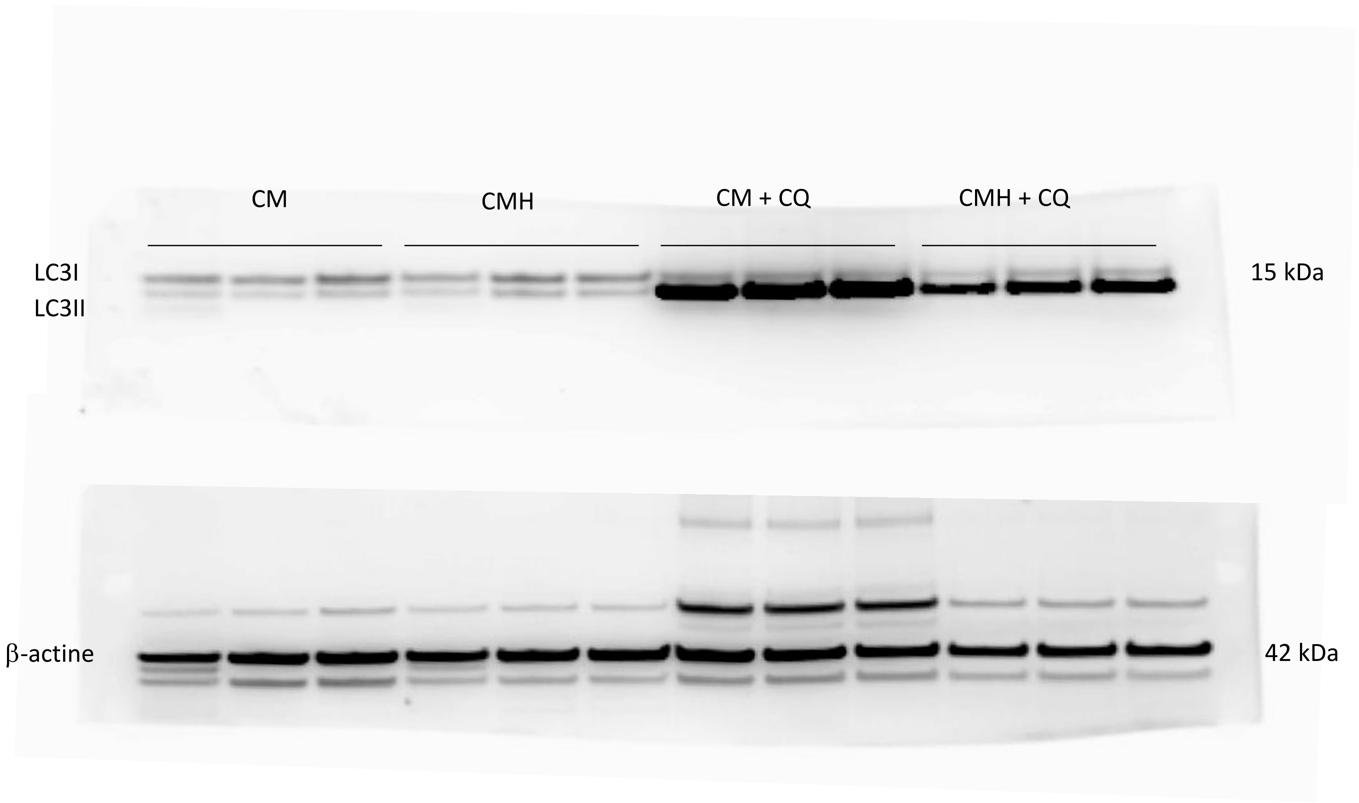

Figure 7B

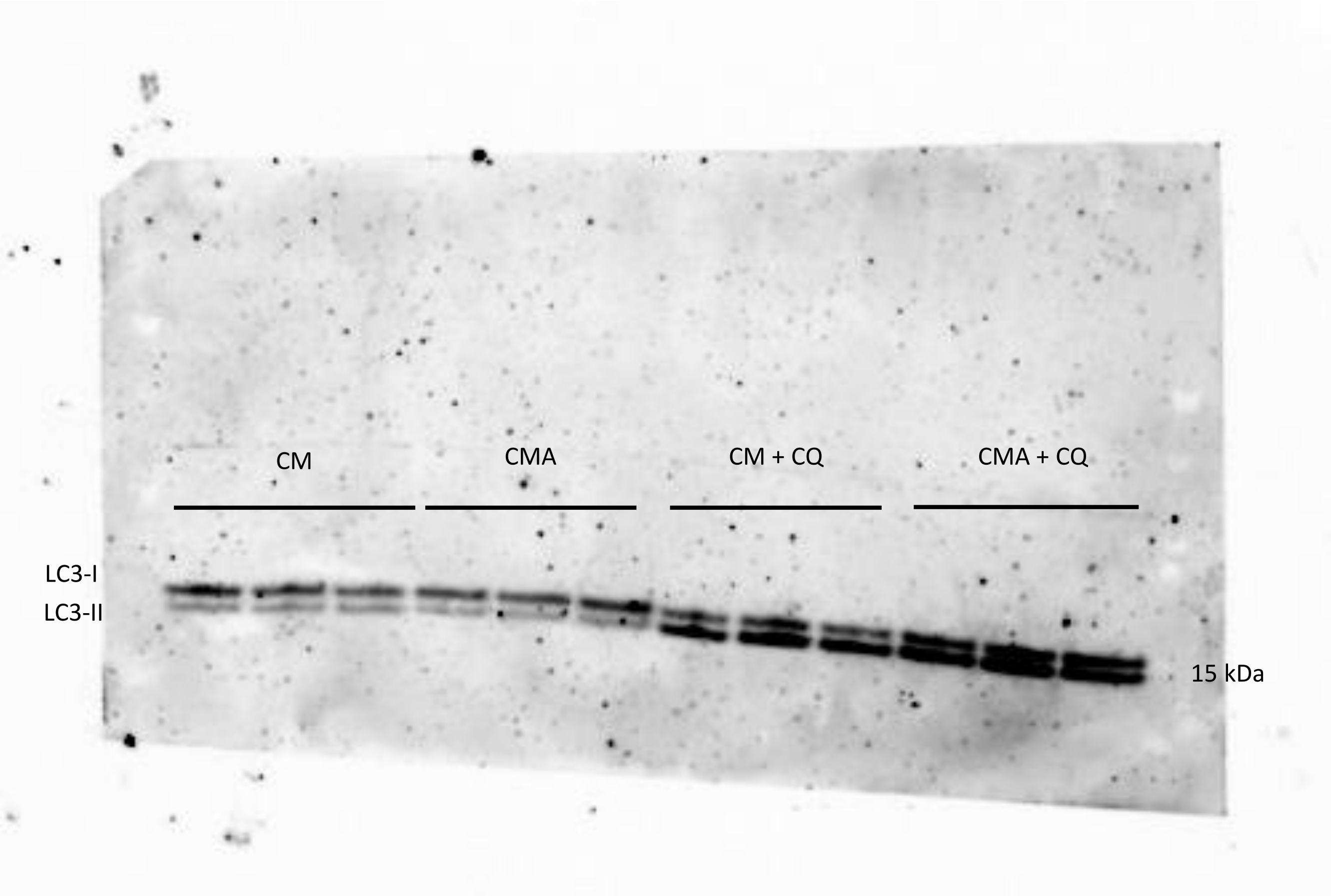
CM
CMA
CM + CQ
CMA + CQ
LC3-I
LC3-II
15 kDa

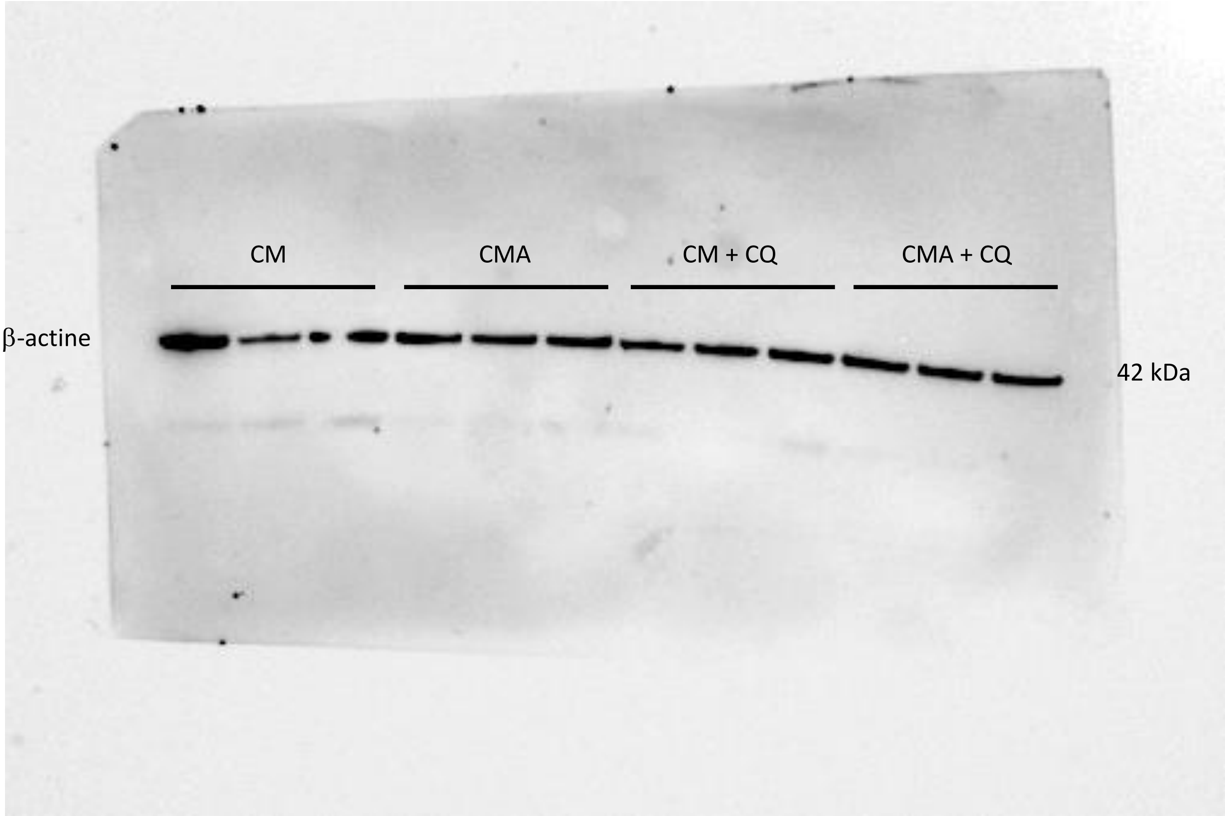
CM
CMA
CM + CQ
CMA + CQ
β-actine
42 kDa

Figure 7C

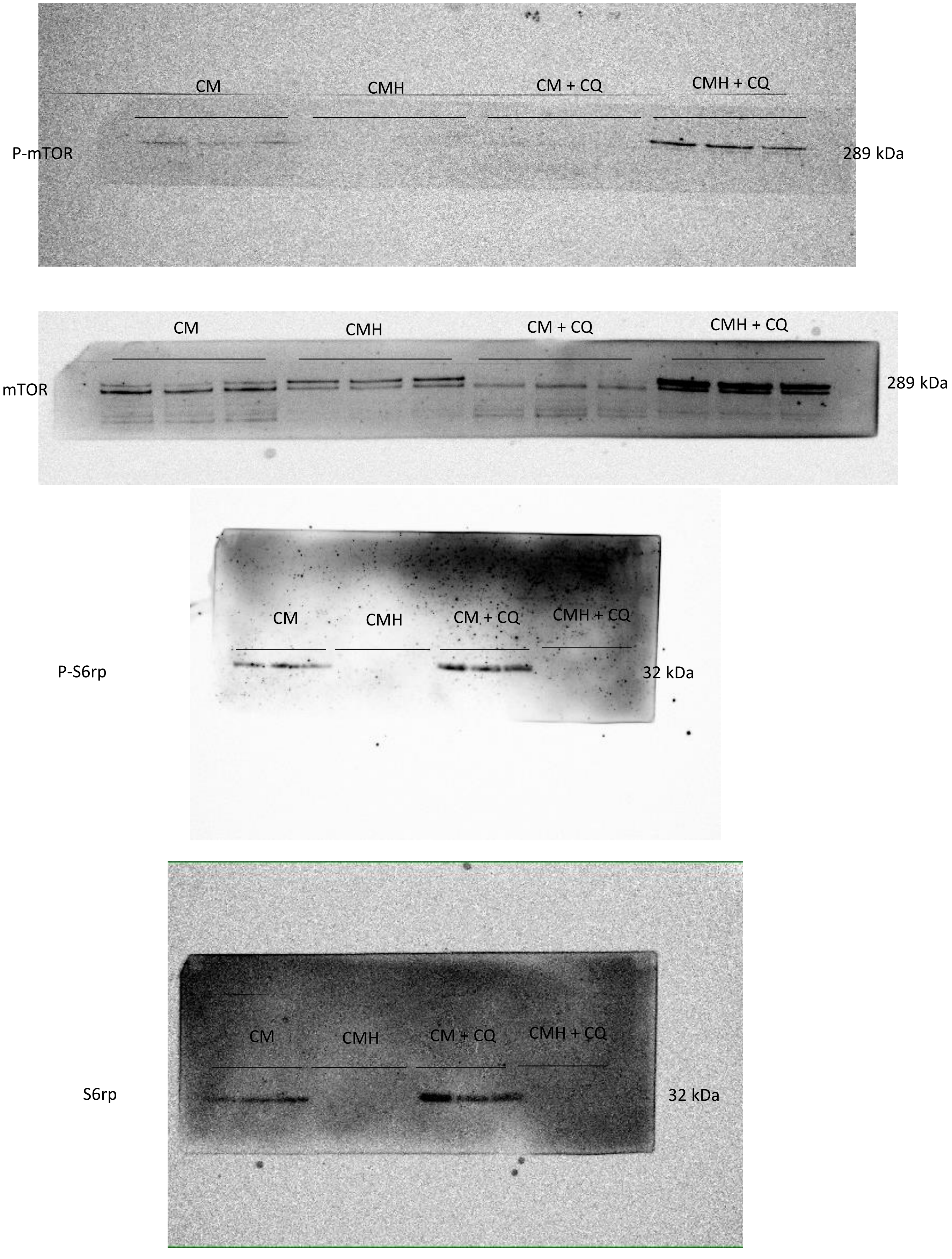
CM
CMH
CM + CQ
CMH + CQ
P-mTOR
289 kDa
CM
CMH
CM + CQ
CMH + CQ
mTOR
289 kDa
CM
CMH
CM + CQ
CMH + CQ
P-S6rp
32 kDa
CM
CMH
CM + CQ
CMH + CQ
S6rp
32 kDa

Figure 7 D

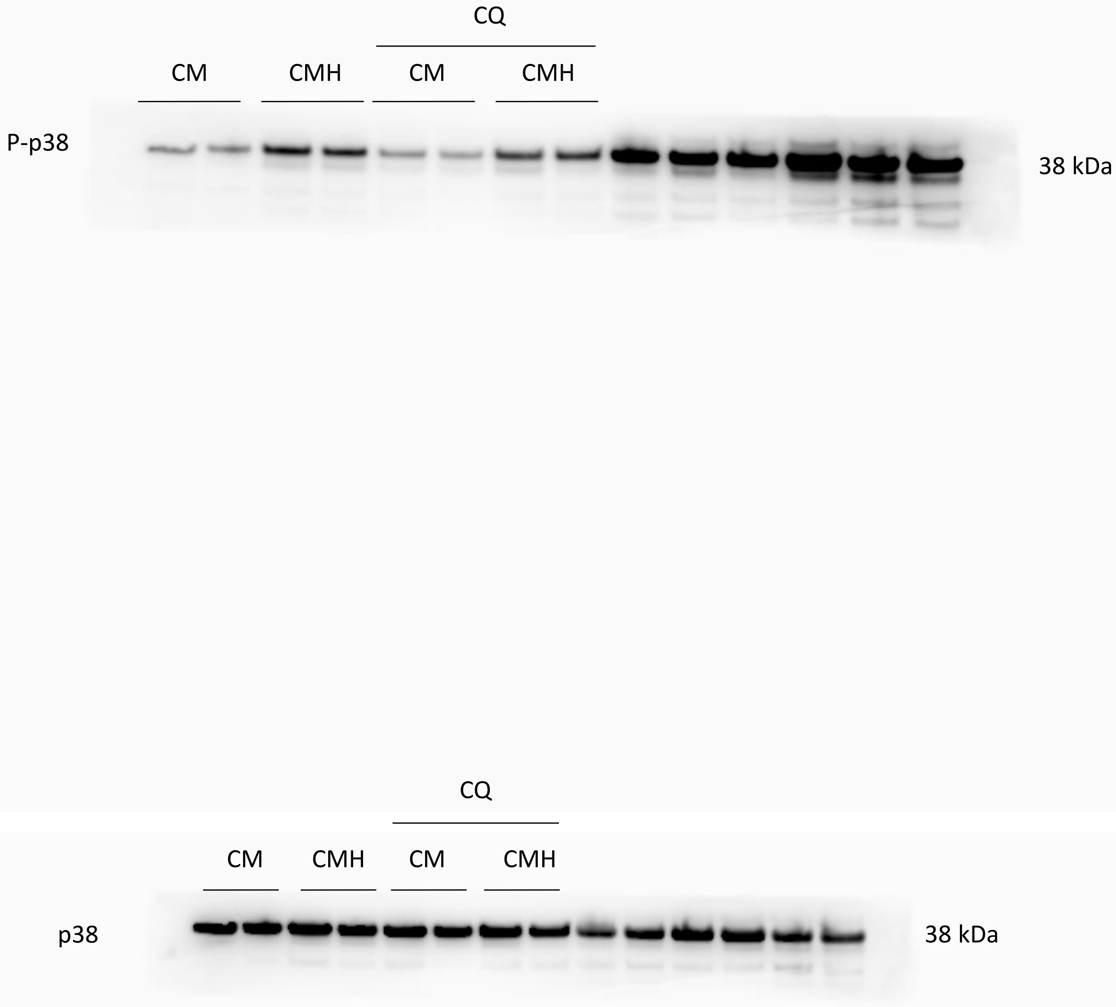

Figure 7E

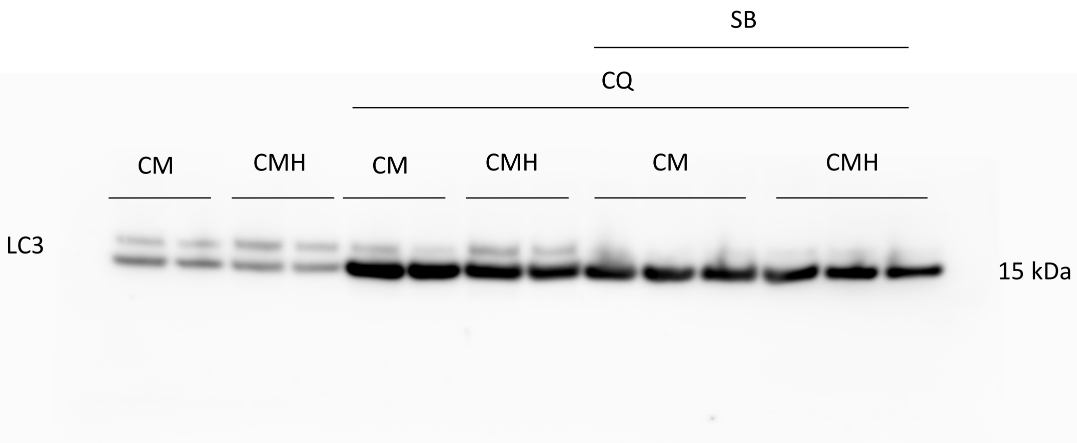
SB
CQ
CM
CMH
CM
CMH
CM
CMH
LC3
15 kDa

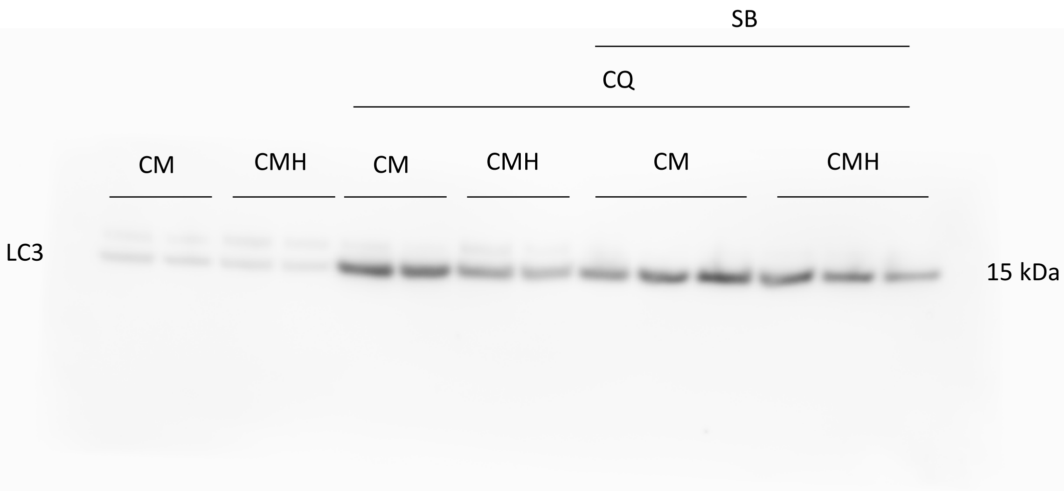
SB
CQ
CM
CMH
CM
CMH
CM
CMH
LC3
15 kDa

Figure 7E

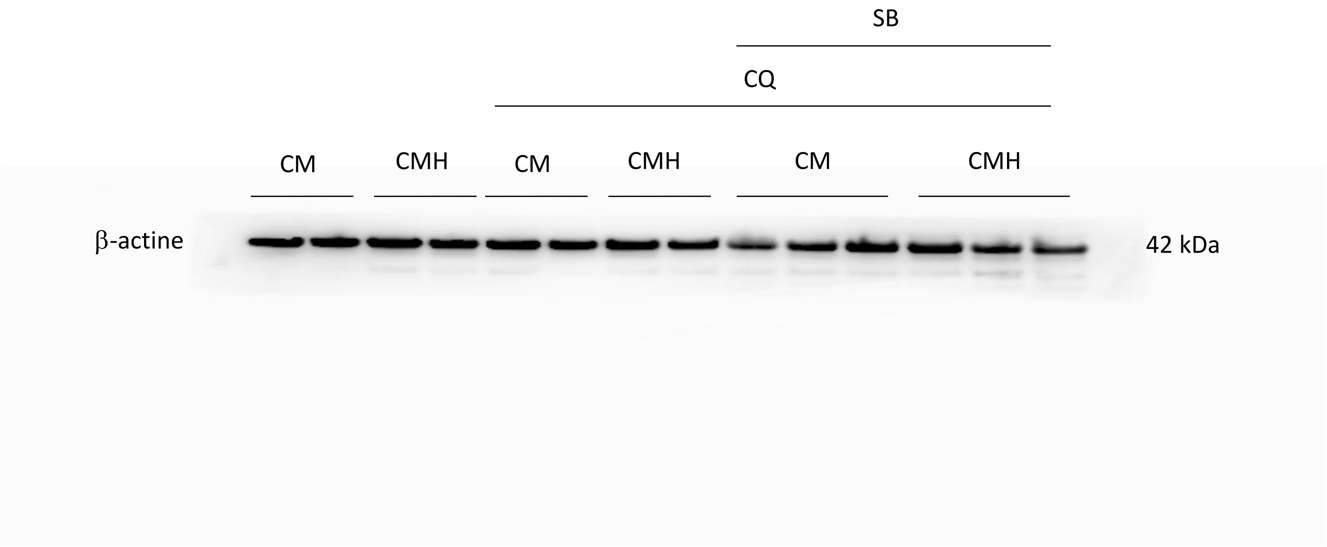

SB
CQ
CM
CMH
CM
CMH
CM
CMH
β-actine
42 kDa

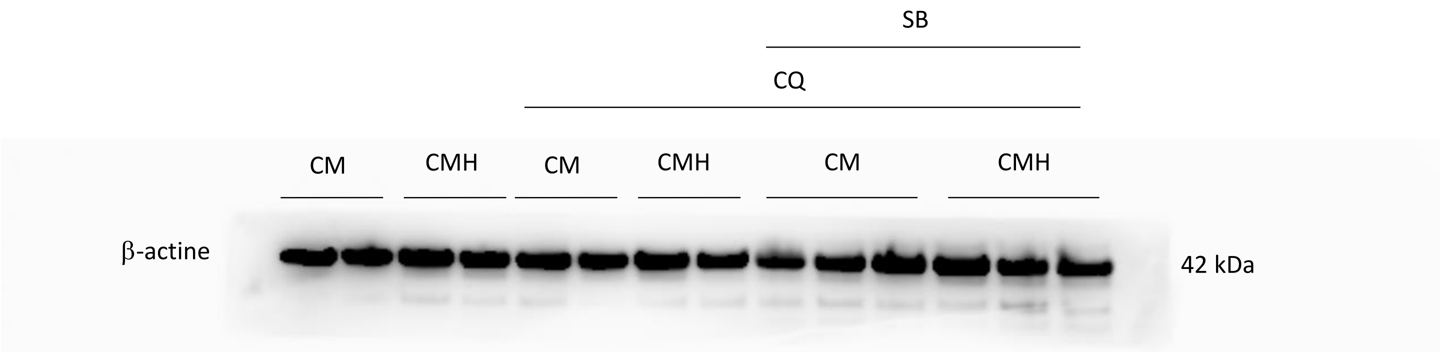

SB
CQ
CM
CMH
CM
CMH
CM
CMH
β-actine
42 kDa
